# *BMC Ecology* image competition 2014: the winning images

**DOI:** 10.1186/s12898-014-0024-6

**Published:** 2014-08-29

**Authors:** Simon Harold, Caspar Henderson, Michel Baguette, Michael B Bonsall, David Hughes, Josef Settele

**Affiliations:** 1 BioMed Central, Floor 6, 236 Gray’s Inn Road, London WC1X 8HB, UK; 2 ᅟ, Twitter: @, casparhenderson; 3 Institut de Systématique, Evolution et Biodiversité, Muséum National d’Histoire Naturelle (MNHN), UMR 7205, Paris, 75005, France; 4 Dept of Zoology, University of Oxford, Oxford OX1 3PS, UK; 5 Department of Entomology and Department of Biology, Center for Infectious Disease Dynamics, Pennsylvania State University, University Park, State College, Pennsylvania, USA; 6 Department of Community Ecology, Helmholtz Centre for Environmental Research-UFZ, Theodor-Lieser-Str. 4, 06120, Halle, Germany; 7 iDiv, German Centre for Integrative Biodiversity Research, Halle-Jena-Leipzig, Deutscher Platz 5e, Leipzig, 04103, Germany

## Abstract

*BMC Ecology* showcases the winning entries from its second Ecology Image Competition. More than 300 individual images were submitted from an international array of research scientists, depicting life on every continent on earth. The journal’s Editorial Board and guest judge Caspar Henderson outline why their winning selections demonstrated high levels of technical skill and aesthetic sense in depicting the science of ecology, and we also highlight a small selection of highly commended images that we simply couldn’t let you miss out on.

## Editorial

“*There is grandeur in this view of life*” [1]. This snippet from *On the Origin of Species* is taken from the famous passage where Darwin expresses his own wonder at the variety of life on earth, stating “*from so simple a beginning endless forms most beautiful and most wonderful have been, and are being, evolved*”.

Although this phrase is more commonly associated with the field of evolution than ecology, the two are undoubtedly intertwined. Perhaps a better quotation could have been taken from earlier in this same passage where he describes a “*tangled bank*” of interacting species. Yet, the proximity of these two ideas in this part of the book speaks a lot about their connectedness.

No discussion about our relationship with the natural world would be complete without a passing nod to Darwin, and the brief sentence above encapsulates nicely how a holistic perspective on ecological processes can elevate the study of natural history into something grand.

This was, in part, our motivation for launching the first *BMC Ecology* image competition in July 2012 [2]. By opening up a perspective on the science of ecology to every researcher on the planet, we hoped to capture a grand perspective of life on earth, its interconnectedness, and its wonder. We hope that, in some small part, we approximated this last year, and we hope to have built on this again.

For this year’s competition, we are very pleased to have writer and journalist Caspar Henderson act as guest judge to choose an overall winner, runner-up and some highly commended images amongst the incredible selection submitted. Caspar has written extensively on a number of scientific and environmental issues, and was most recently shortlisted for the Royal Society Winton Prize for Science Books for his *Book of Barely Imagined Beings: A 21st Century Bestiary*[3]. He is now working on *A New Map of Wonders*.

His nominated charity of choice for this year is Trees for Life [4], a foundation established in 1989 with the aim of restoring Scotland’s ancient Caledonian Forest. The ultimate aim of the charity is to raise enough money to restore a region of 1,000 square miles of mountains and glens with re-wilding of native flora and fauna. Currently an area that has suffered much environmental degradation, the project aims to restore native woodland cover and eventually wildlife including wild boars (*Sus scrofa*) and beavers (*Castor fiber*).

As well as our guest judge, the Editorial Board of *BMC Ecology* were also on hand to pick their favourite category winners for each section, blinded to the identity of each entrant. More than 300 individual images were submitted to the competition this year, depicting some form of ecological interaction from every continent on earth.

We hope you enjoy them.

## Winning images

“*How have all [the] exquisite adaptations of one part of the organisation to another part, and to the conditions of life, and of one distinct organic being to another being, been perfected?*” So asked Charles Darwin in 1859, articulating one of the central questions of ecology half a dozen years before the term was even coined. How could such “*beautiful co-adaptations*” have arisen in every part of the organic world?

Darwin’s answer, subtle and complex, helped make the discipline of ecology possible. And for more than 150 years those who call themselves ecologists have been unpicking astonishing complexity and pattern within living systems. There may be (in the timeworn and not always helpful phrase) a “*struggle for existence*” in the living world; but there is also so much more. In its stupendous productivity and capability to generate variation, Life presents a spectacle for which a description coined by Pliny the Elder more than 1800 years before Darwin is no less apt: “*the great variety of nature at play*” [5].

All those who have taken part in this competition have produced work that contributes to a great tradition of enquiry and analysis that is contemporary ecology. The best work often shows that new phenomena - sometimes startling, sometimes beautiful and sometimes both - are always there to be found with the keenest eye, the sharpest act of attention. At a time of exceptionally rapid change to the non-human systems upon which all life depends, such work was never more important, and shows that while we may have great cause for concern, there is also no end to marvels.

So congratulations to the winner Petra Wester and runner-up Letizia Campioni, and all those whose photographs are highly commended. Your work shows particularly high levels technical skill in photography and/or aesthetic sense (and often both), as well as illuminating fascinating and diverse questions. But well done all who took part. This world is not all about winning competitions. We are all hear to wonder, and learn.

### Overall winner

**Figure 1:**
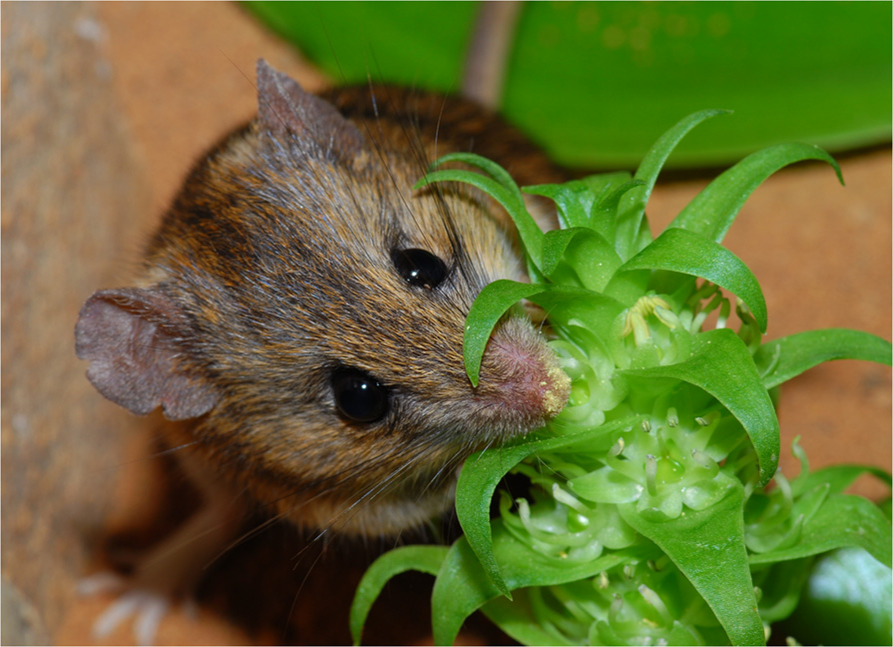
**Overall winner.** “A Namaqua rock mouse (*Aethomys namaquensis*, Muridae) getting dusted with pollen of the Pagoda Lily (*Whiteheadia bifolia*, Hyacinthaceae) while lapping nectar at the flowers.” Attribution: Petra Wester.

Petra Wester (Institute of Sensory Ecology, Heinrich-Heine-University Düsseldorf, Germany) (Figure 1):

At first glance, this image of a Namaqua rock mouse (*Aethomys namaquensis*, Muridae) getting dusted with pollen of the Pagoda Lily (*Whiteheadia bifolia*, Hyacinthaceae) might not appear to be particularly striking. But this is an image that is much more than it at first appears. In contrast to last year’s winning image, of an evolutionary adaptation driven by the avoidance of death, this year’s winner offers a fascinating window into a highly unusual evolutionary game in which mutual benefits aid the struggle for survival and reproduction. We tend to think of insects, and occasionally birds, when we think of pollination ecology, and this winning image therefore serves as a reminder of the variety of different ways in which nature can converge on the “beautiful co-adaptations” that so fascinated Darwin:

“*The picture was taken at night during a study of the pollination ecology of the Pagoda Lily. For the first time nocturnal rodent pollination was observed and photographed under natural conditions in the Northern Cederberg area of South Africa. Field studies and experiments showed that the flowers of* Whiteheadia bifolia *are visited at night by Namaqua rock mice. The mice were observed licking the extremely viscous nectar while being dusted with pollen around the snout and touching the stigmas of the flowers without destroying them.* W. bifolia *has characters of the rare rodent pollination floral syndrome such as visually inconspicuous, bowl-shaped flowers near the ground, stiff stamens, easily accessible nectar and a weak nutty scent. No other visitors were observed during the day or night.”*

### Overall runner-up

**Figure 2:**
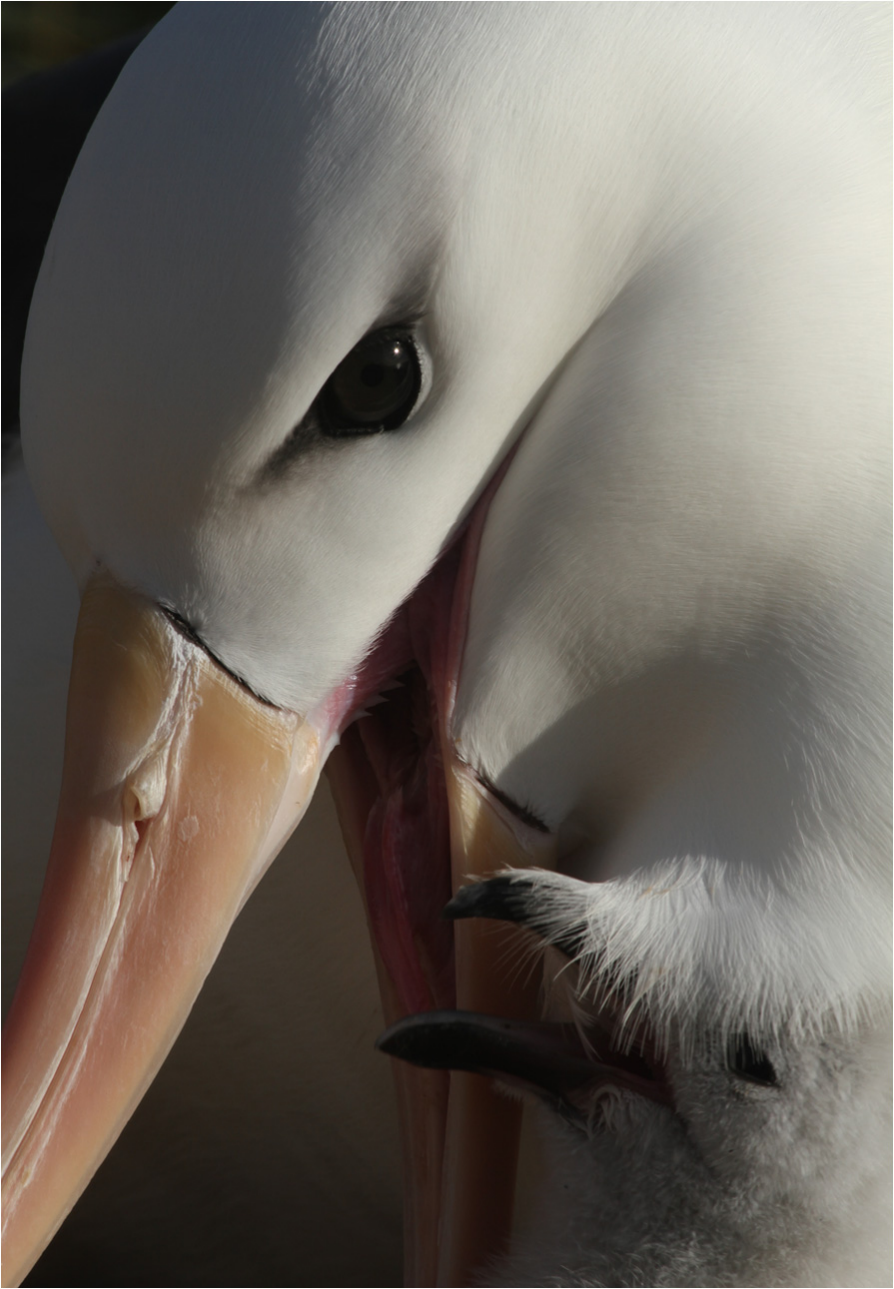
**Overall runner-up.** “Black-browed albatross (*Thelassarche melanophrys*) and chick on New Island (North-west Falkland I.). One aim of my work is monitoring the demography of these populations. Our objective is to follow the whole population during the different phases of the breeding season recording breeding pair success and fledgling success.” Attribution: Letizia Campioni.

Letizia Campioni (Eco-Ethology Research Unit, ISPA, Portugal) (Figure 2):

Parental care is at the heart of this wonderful portrait of an adult Black-browed albatross (*Thelassarche melanophrys*) feeding its chick. Offspring rearing comes in many guises in the animal kingdom, defined by a complex interplay of trade-offs between investment and return. Many arthropods, for example, produce a huge abundance of offspring, left to fend for themselves from the point of birth in the hope that some will survive. In stark contrast, long-lived animals such as albatross—who often live to around 70 years of age—invest heavily in the survival of a single chick each year, regurgitating food until they are ready to fledge. The gorgeous detail of this image also serves to highlight fascinating adaptations to life foraging on the wing – the pronounced nostril, or naricorn, that guides saline solution from the salt gland, and the tooth-like structures at the base of the bill—all features for survival in a maritime environment. Although currently not endangered, ever declining populations of these majestic creatures is a cause for concern, particularly in light of evidence that human practices may partly be to blame [6]. The work of ecologists like Letizia Campioni in monitoring and understanding these populations can therefore not be underestimated:

“*My field of research is focused on the study of long-lived pelagic seabirds. Specifically I am working on Black-browed albatross (*Thelassarche melanophrys*) nesting in dense colonies on New Island (North-west Falkland I.). One aim of my work is monitoring the demography of Black-browed albatross populations. Our objective is to follow the whole population during the different phases of the breeding season, recording breeding pair success and fledgling success.”*

## Section winners

### Behavioural and physiological ecology

**Figure 3:**
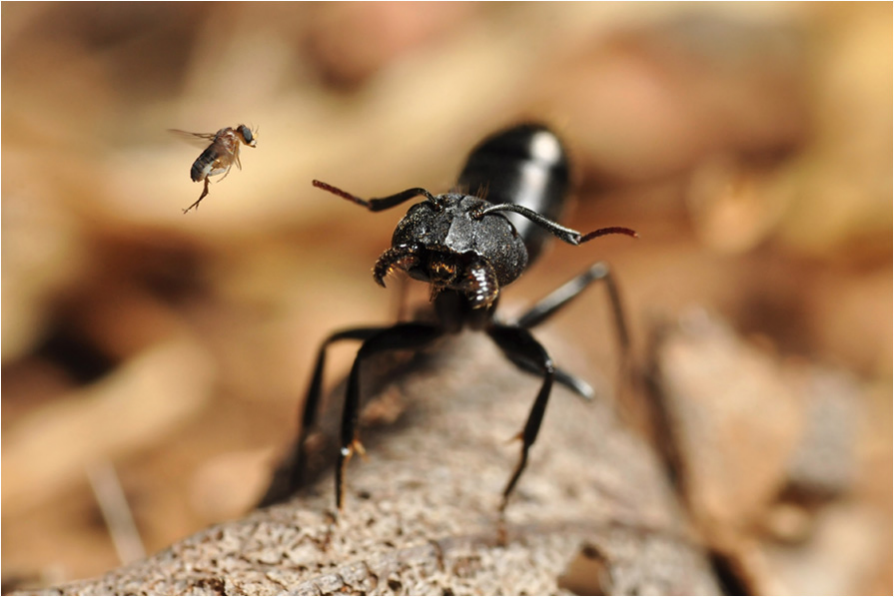
**Winner:*behavioural and physiological ecology.*** “*Camponotus morosus* ant being attacked by a parasitoid phorid fly (Diptera: Phoridae). At the moment of the attack the ants were involved in a intra specific fight between two different ant nest and presumably the fly detected the ants because of the alarm pheromones released during the fight.” Attribution: Bernardo Segura.

Of all the section categories, the *Behavioural and Physiological Ecology* section was by far the most popular in the competition, representing a full third of all images submitted. This may be due in part to a tendency for the eye to be drawn to portraits of single species, rather than viewing whole communities or ecosystems of interacting organisms. It may also reflect that these types of images are inherently less difficult to capture. But this is not to take anything away from this category, as the winning image from Bernardo Segura of the Laboratory of Fragmented Landscapes (LEAF) at the University of Chile attests (Figure 3). This strikingly dynamic moment in which a *Camponotus morosus* ant comes under attack from a parasitoid phorid fly is captured in astonishing detail, as Segura explained in the accompanying text:

“*At the moment of the attack the ants were involved in a intra specific fight between two different ant nest and presumably the fly detected the ants because of the alarm pheromones released during the fight. The phenomena is described in Segura & Brown 2014*[7]*.”*

This moment, the first report of a parasitoid attacking *Camponotus morosus*, or any *Camponotus* ant in Chile, clearly made an impression on Section Editor and judge David Hughes, himself an ant ecologist:

“*Obviously a very beautiful photo but I enjoyed how the description detailed the competition between ants and the chemical ecology; and how the parasitoid was taking advantage of this. It is really impactful because it highlights how organisms have multiple competing ecological pressures (in this case competition for food/space and parasite pressure).”*

### Community, population and macroecology

**Figure 4:**
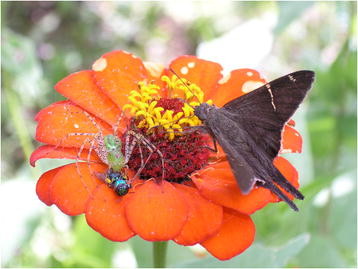
**Winner:*community, population and macroecology.*** “A crab spider on a flower preying upon a euglossine bee, while a butterfly looks for nectar, taken on the slopes of Cerro Chucantí, Darién Province, in eastern Panama. Note, the bee had already filled his ‘saddle bags’ with pollen before being attacked by the spider. The petals on the far side of the flower also appear to be succumbing to some kind of blight, perhaps due to a fungus.” Attribution: Andrew J. Crawford.

Few images submitted to the competition this year managed to depict as many ecological interactions in one frame as that submitted by Andrew J. Crawford of the Smithsonian Tropical Research Institute in Panama, and Universidad de los Andes in Bogotá, Colombia (Figure 4). Taken on the slopes of Cerro Chucantí, the highest point in the Maje Mountain Range of eastern Panama, the image shows a crab spider preying upon a euglossine bee, while a long-tailed skipper butterfly looks for nectar on the same flower. There is so much happening in this one image, that it is easy to miss some of the finer details that make this shot so special, as Dr Crawford explains:

“*Note, the bee had already filled his ‘saddle bags’ with pollen before being attacked by the spider. The petals on the far side of the flower also appear to be succumbing to some kind of blight, perhaps due to a fungus. Thus, this photo shows multiple ecological processes taking place simultaneously on one small, beautiful flower.*”

Not only do the vivid hues of flower, spider and pollen combine beautifully with the iridescence of the stricken bee, taken as whole the image captures a fleeting snapshot in miniature of the many community-level interactions that take place on other flowers, other habitats and other ecosystems on a global scale.

### Conservation ecology and biodiversity research

As a proportion of all the images entered into this year’s image competition, marine ecosystems were perhaps the most underrepresented, with only around 5% of entries depicting an ecosystem that covers some 70% of the globe. This may partly reflect the logistical difficulties of taking images underwater, but there is also a good chance that it may be due to a more widespread disconnection of humans with this type of environment. As one of the Co-ordinating Lead Authors (CLAs) on a chapter in the latest Intergovernmental Panel on Climate Change report [8], Section Editor Josef Settele is well aware of the threat posed to these marine habitats, which often reflect some of the richest biological diversity found on the planet:

“As the year 2014 experiences a highlight in the publication of the new IPCC report, and as corals are highlighted there as being among the most threatened systems under climate change, I felt that the example presented here sets an important spotlight on what is at stake and is not accessible (literally and thus emotionally) to the vast majority of people. Furthermore biodiversity hotspots like the Philippines and its surrounding seas deserve far more attention.”

**Figure 5:**
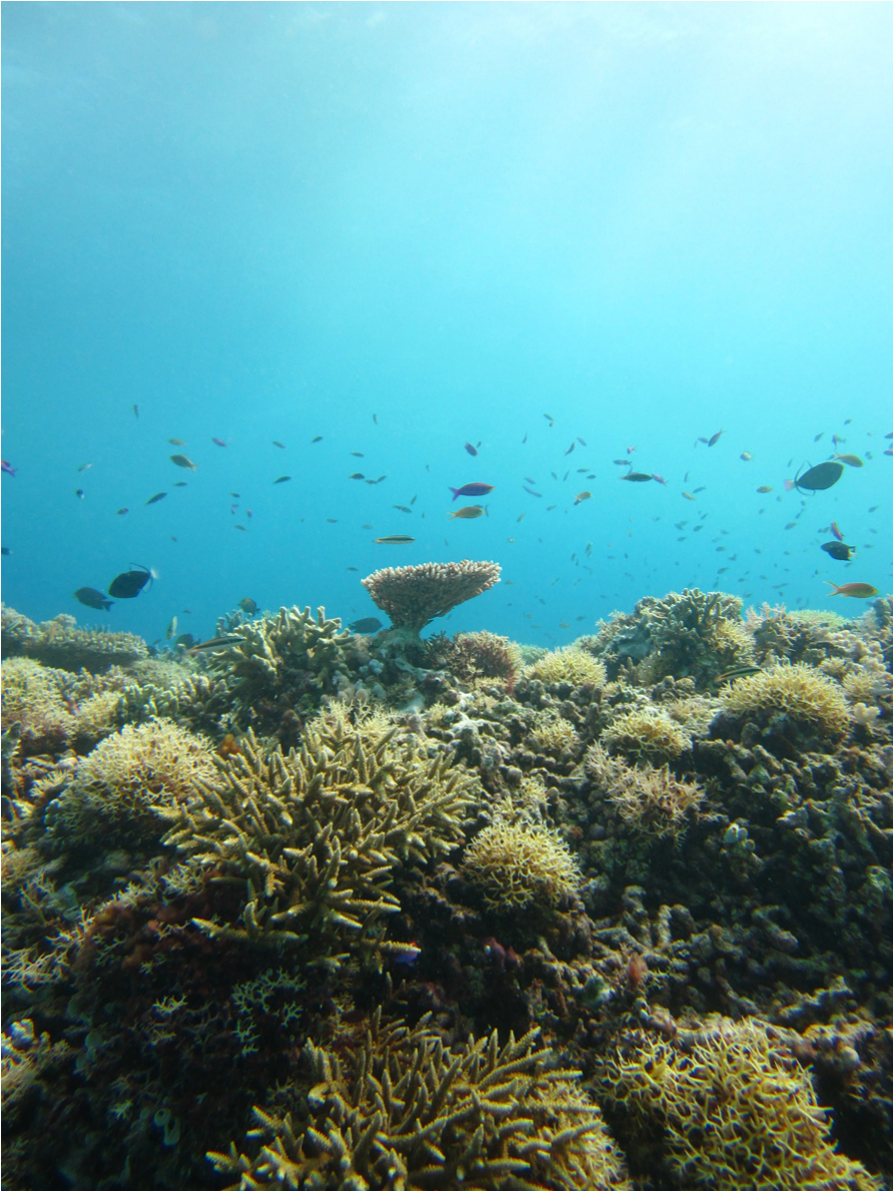
**Winner:*conservation ecology and biodiversity.*** “The image was taken on a Catlin Seaview Survey expedition to Tubbataha Natural Park of the Philippine waters of the Sulu Sea in the heart of the Coral Triangle. A young, tabulate Acroporid coral can be seen reaching its branches toward the sun to fuel its Symbiodinium algal symbionts with a plethora of reef fishes in the background. It is the marine equivalent of a young tree shooting skyward to fulfil its ecological role in a forest. Corals, branching Acroporids especially, contribute greatly to habitat complexity, or rugosity, of reefs increasing the microhabitats for other reef creatures.” Attribution: Catherine Kim.

University of Queensland PhD student Catherine Kim’s depiction of coral reef biodiversity was taken as part of the Catlin Seaview Survey expedition to Tubbataha Natural Park of the Philippine waters of the Sulu Sea (Figure 5). A key aim of surveys such as this is to bring this largely unseen diversity to the attention of the public, through the use of easily accessible interfaces based on software that is widely used by the general public, as Catherine explains:

“*As the center of marine biodiversity, there are over 350 species of coral and 500 species of fishes in Tubbataha. A young, tabulate Acroporid coral can be seen reaching its branches toward the sun to fuel its Symbiodinium algal symbionts with a plethora of reef fishes in the background. It is the marine equivalent of a young tree shooting skyward to fulfil its ecological role in a forest. Corals, branching Acroporids especially, contribute greatly to habitat complexity, or rugosity, of reefs increasing the microhabitats for other reef creatures. In the face of a changing climate and increasing human impacts coral reefs face mounting challenges for their survival which makes the protection and conservation of these important ecosystems even more vital. The Catlin Seaview Survey is dedicated to monitoring the world’s changing coral reefs and communicate the state of reefs visually to the world via Google Oceans Underwater Streetview. The goal is to reach the greater than 99% of the population that does not SCUBA dive to share these unique ecosystems and incite the desire to conserve coral reefs. It is an ambitious project and necessary if these special marine habitats are to have a hopeful future.”*

### Landscape ecology and ecosystems

**Figure 6:**
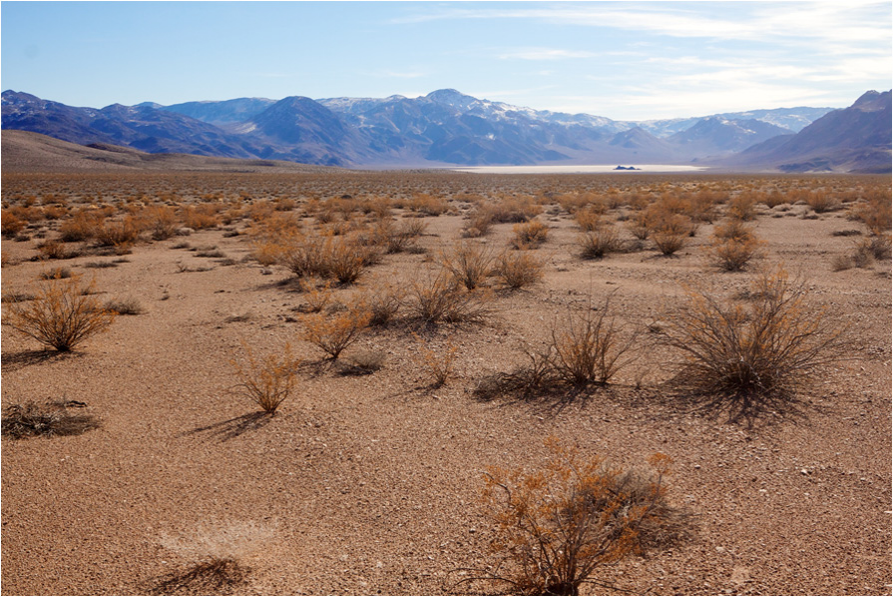
**Winner:*landscape ecology and ecosystems.*** “An endorheic basin in Death Valley, California. Although annual precipitation rarely exceeds 100 mm/yr, a small number of plants are able to survive on the gravely slopes of the valley and on the muddy lakebed. Thousands of years ago this valley would have been far more wet and lushly vegetated.” Attribution: Benjamin Blonder.

Landscape ecology is all about space. Specifically, how the spatial relations between different organisms and their environment affects their patterns of distribution and abundance. Capturing the essence of this discipline visually is not easy, and so Benjamin Blonder from University of Arizona should be congratulated for his image of vegetation growing in a strangely uniform pattern in an endorheic, or closed-drainage, basin in Death Valley, California (Figure 6). Although this ecosystem would appear to be almost denuded of biological diversity, Dr Blonder, a runner-up in last year’s image competition, reminds us that ecosystems are often anything but static entities:

“*Although annual precipitation rarely exceeds 100 mm/yr, a small number of plants are able to survive on the gravely slopes of the valley and on the muddy lakebed. Thousands of years ago this valley would have been far more wet and lushly vegetated.”*

Section Editor Michel Baguette was equally impressed by the variety of ecosystems that the image depicts in this inhospitable part of the world, and the adaptations that species must exhibit in order to survive there:

“I am puzzled by the regular organization of the vegetation, typical of plants growing on a poor soil. I also like the juxtaposition of different ecosystems (desert, lake, mountains).”

### Theoretical ecology and models

**Figure 7:**
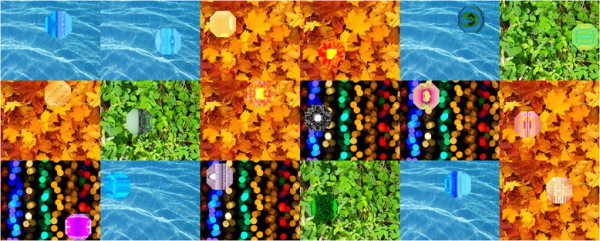
**Winner:*theoretical ecology and models.*** “A collage of some of the best and most interesting results from a series of computational simulations of the coevolution of prey camouflage and predator vision.” Attribution: Kyle Harrington.

When it comes to communicating the science of ecology to non-specialists, the theoretical side of the discipline is often overlooked in favour of studies involving charismatic species. This is perhaps understandable, but belies the fact that so much of ecology is underpinned with strong theoretical and mathematical foundations. In the light of this, we were delighted to receive this image from Kyle Harrington, a postdoctoral scientist at Brandeis University, Dynamical and Evolutionary Machine Organization in the US, which was eventually chosen as the Section winner for *Theoretical Ecology and Models* (Figure 7). This striking collage of public domain images with some of the most interesting results from a series of computational simulations on the coevolution of prey camouflage and predator vision is not only aesthetically pleasing, but displayed a wonderful sense of creativity beyond only the output of a simulation. Section Editor Mike Bonsall was impressed by this combination, which we hope will be a benchmark for entries to this Section in future competitions:

“*It is visually appealing and shows the wide array of camouflage predator vision patterns predicted using a genetic algorithm. Super neat set of pictures....and some nice science!”*

For those that are still unsure of the theoretical underpinnings of the model, you can read more in a recent publication by Dr Harrington [9], which he explains a little more about here:

“*Prey patterns are produced by a developmental process inspired by chemical morphogenesis, using a technique called genetic programming. Predators use a model of visual attention inspired by human neurophysiology. We simulate the coevolutionary interactions between predators and prey, where predators are rewarded for detecting prey, while prey are rewarded for avoiding predation. By simulating coevolution over many generations we observe the transition from patterns with no prior bias for their environmental context, to the emergence of camouflaged prey patterns.”*

### Editor’s pick

**Figure 8:**
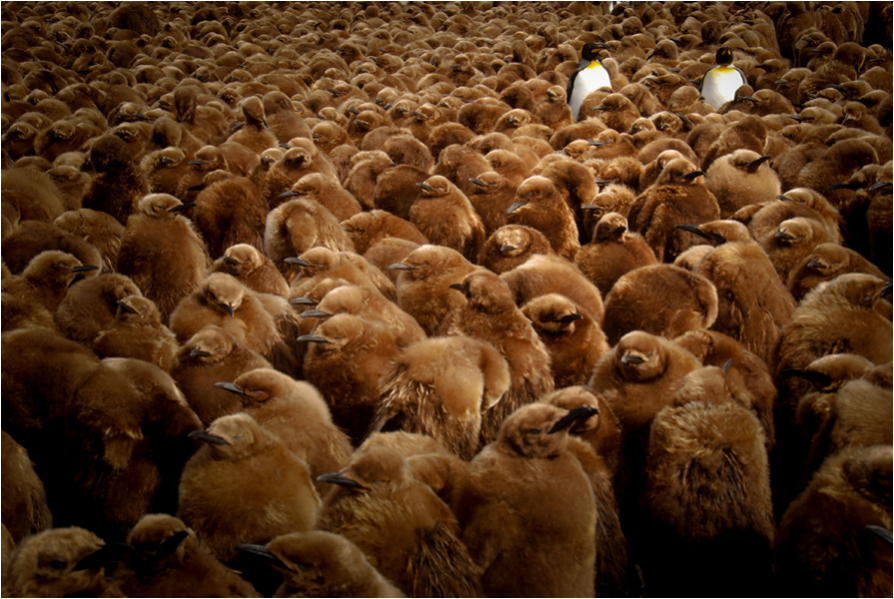
**Winner:*editor’s pick.*** “King penguin chicks (*Aptenodytes patagonicus*) huddle together in huge crèches of several thousand chicks.” Attribution: Laetitia Kernaleguen.

Some biomes are harsher than others, and conditions don’t come much more hostile to life than eking out an existence in Antarctica and the sub- Antarctic islands. King penguins, for example, endure extremes of cold in addition to long foraging trips to ensure adults and offspring alike are fed well enough to endure the long winters. This beautifully composed image by previous category winner Laëtitia Kernaléguen from Deakin University captures the adaptation of an iconic species in this region to both the extreme cold, and the threat of predators (Figure 8). The indeterminable scale of the image is what gives it such power – as viewer, we can’t know how many juvenile penguins have congregated in this group, but we know their numbers are vast. Adult birds stand in stark contrast, their morphology adapted more to underwater hunting than thermal insulation, their vast outnumbering by juveniles only serving to heighten the sense of burden and overwhelming responsibility that parents must have to provide for their young. The sense of serenity this picture conveys is perhaps what is most surprising—and heartening—given the extremes of hardship they must endure.

“*Catch me if you can… discern me! Winter is a hard time for king penguin chicks (*Aptenodytes patagonicus*). Left alone for months while their parents are gone fishing hundreds of miles away, they have to struggle against the cold, the snow and above all, the fascinating giant petrel. How to fight, while already starving, against a giant bird that daily comes to the colony to check its meat safe? Penguins huddle together in huge crèches of several thousand chicks. United we stand, divided we fall.”*

### Highly commended

The natural world can sometimes seem a brutal place, but there is also beauty in abundance. Not only in the colour, form and variety of organisms but in their relations, in something more than just aesthetics. Of course, as viewers we can’t help but see these images through anthropogenic eyes—but that’s ok. Each of the facets that makes an image wonderful, shocking, or awe-inspiring will have been the result of the ecological and evolutionary processes that place them in our view, and it is the skill of the person that captures this moment that makes it so special.

So it is that in selecting only those images that struck a chord with the judges, we know that much astonishing variety has had to be discarded. The following selection of highly commended images is therefore an attempt to provide a flavour of the extraordinary quality of entries this year, and hint at the complexity of ecological processes happening on earth. From the sunlight bursting through the wings of swallowtail butterflies crowded around a mineral deposit [Additional file 1], to the incredible fluorescent orange of a bacterial/cyanobacterial crust community in an anchialine pond of the Hawaiian Islands [Additional file 2], spectacular colouration has always been a big part of what makes a successful image in nature. That the latter is formed by micro-organisms is particularly pleasing since, relative to their ubiquity in nature, this group remains largely forgotten in depictions of the natural world.

A sense of sheer joy is coupled with the ever-present danger posed by predators in a less-colourful, but no less spectacular, image of spirited gelada baboons as the sun sets on the Ethiopian Highlands [Additional file 3]. This species roams in large groups with complex social structures, and it is always difficult not to see some human-like qualities among these populations. This is only exacerbated if we examine our closer relatives, the great apes, in more detail. A wonderful, colourful portrait of a female chimpanzee and her infant demonstrates this well, as the infant gazes back at us with knowing curiosity [Additional file 4]. Harvard University ecologist Alain Houle took the image 25 metres up a tree in Kibale National Park, Uganda, as part of his postdoctoral research into the relationship between contest competition and the nutritional quality of wild fruits which, as he explains, was by no means a straightforward task: “*It was my desire to climb the tree crowns to observe the chimpanzees’ behaviors in details, I was seriously warned by all primatologists and authorities of the National Park that this attempt would be risky (climbing tropical trees) and very dangerous (because chimpanzees would respond aggressively to my presence in the same fruit tree)….I proved during my research study that wild chimpanzees that are habituated to human observers on the ground are tolerant to, and most importantly do not alter their natural behaviors in the presence of, human observers in the canopy.”*

Hands-on parental care is not just restricted to the higher primates, and there is certainly a great deal of variety of care practices in nature that makes for interesting imagery. Sphecid wasps such as this mating male and female *Sphex latreillei* [Additional file 5], adorned in their luxurious red coats, continually provide fresh food for their developing larvae who remain out of sight, buried in a burrow [10]. In contrast, many species of Hemipteran insects, characterised by their sucking mouthparts, will leave their offspring to fend for themselves after eggs are laid on a host food plant [Additional file 6].

One primate that takes a lighter attitude to its offspring is the slender loris, its surprised gaze nicely captured by Sayantan Das of University of Mysore [Additional file 7]. The juvenile in this picture has been left in a ‘parking spot’ in a hidden location by its mother, who leaves to forage for food in the night. Lorises are well adapted to a nocturnal lifestyle, using their large eyes to hunt for insect prey. One hopes that the juvenile in this photograph soon recovered from the inconvenience of staring directly into the barrel of the camera’s flash gun. Night hunters make a habit of keeping well hidden, especially during the day, as these curious Spotted Owlets (*Athene brama*) roosting in the hollow of a tree attest [Additional file 8].

The ability to hide in plain sight is something that the arctic hare (*Lepus arcticus*) does best, blending into their snowy background with a white winter coat to evade predators. However, the transition between seasons can leave individuals vulnerable to predation until their coats change to match their conditions [Additional file 9]. The ancient arms race between predator and prey was a popular subject in this year’s competition, captured with wonderful depth of field in this image of an oriental garden lizard contemplating its next meal [Additional file 10]. Parasitism was a less prevalent subject matter, but an image that really stood out in this context was of two water fleas (*Daphnia magna*) and their differing fates [Additional file 11]. Nina Scholz of the University of Konstanz, whose work on Daphnia has been previously published in *BMC Ecology*[11], explains the impact that parasitism has on these tiny crustaceans: “*One of these villains is* Pasteuria ramosa*, a bacterial parasite castrating its host after successful establishment…The comparison of a healthy (left) and a heavily infected (right) female reveals the consequences of parasite invasion: while the uninfected animal holds developing neonates in its brood chamber, this chamber is empty in the infected animal [which] turn reddish in color. After the death of the host, spores will be released from the carcass and consequently be ingested by new hosts, completing the horizontal transmission and starting a new life cycle.”*

Inter- and intra-specific conflict in many guises was well represented in a number of categories, and the judges were particularly drawn to a somewhat uneven contest between hippos in Amboseli National Park, Kenya [Additional file 12], and a much more static—but no less illustrative—image of the struggle for space between zoanthids and Barnacles in this intertidal community [Additional file 13]. Space may not be at a premium on the Kenyan savannah, but for the wildebeest depicted in this spectacular picture-postcard landscape from Graeme Shannon of Colorado State University [Additional file 14], avoiding predators is very much a priority. Another highly-commended image from the lens of Dr Shannon makes this gruesomely, awesomely clear [Additional file 15].

Lions play a crucial role as top predators in keeping populations of herbivores in check, and while this dynamic image may at first glance show lions at their most fearsome, their prey was in fact bait, luring them toward wildlife monitors: “*This image was captured in Pilanesberg National Park, South Africa during a management operation to dart and anesthetize the male lion on the left of the photo… Maintaining a healthy population of lions requires collecting detailed data on genetics, body weight, condition and disease.*”

Crucial monitoring work like this clearly struck a chord with the judges, with our Section Editors also impressed by another of overall runner-up Letizia Campioni’s work on populations of Black-Footed Albatross [Additional file 16]. In contrast to the portrait of Albatross and chick (Figure 2), this category winner exhibited more of a reportage style – something that clearly caught the eye of Section Editor David Hughes: *“Most of the images were behavior; but this one captured quite a number of excellent elements. First was behavior of course, but then there was field work and mapping and the collection of samples. It was a pleasing mix and impactful as it communicates what many of us do out in the field.”*

Another image of work in the field was highly commended by *Conservation Ecology and Biodiversity Research* Section Editor Josef Settele, depicting the work of conservation scientists in monitoring the spread of viral diseases in bats and how they may impact on humans [Additional file 17]. Here, he explains the importance of this type of research: *“The picture shows real enthusiasm for the conservation of biodiversity, as well as realism by not ignoring the downsides this sometimes is believed to have. The researcher is well prepared technically and very dedicated mentally to tackle important problems, like the spread of diseases through organisms which are endangered in order to find real solutions for conflicting interests.”*

Other depictions of man’s contact with the natural world were equally impressive, such as a creative montage of images undertaking field work in coastal areas of western India [Additional file 18], and a distressed young bird in the hands of its rescuer [Additional file 19]. A charging horde of bright blue soldier crabs (*Mictyris longicarpus*) marching through the aerial roots of the mangrove *Avicennia marina* also serves as a timely reminder of the benefits that a closer association of man with nature can bring [Additional file 20]. Matthew Nitschke of the University of Queensland explains “*Soldier crabs roam across mudflats, feeding on detritus and the microphytobenthos. Due to their behavioural and feeding ecology, they can potentially serve as important biomarkers. An analysis of soldier crab physiology can provide information on heavy metal pollution in compromised waterways. This will prove especially useful when conventional water sampling methods cannot detect low concentrations of pollutants.”*

The rapid pace of human population expansions and their inevitable collision with the natural world was the focus of a number of entries, particularly in the *Conservation Ecology and Biodiversity Research,* and while many served as a salutary warning, reminding the viewer of the negative impact of the Anthropocene era, two images in particular stood out for the positive message they conveyed. The first depicts a stricken Cory’s shearwater fledgling (*Calonectris diomedea*) on a tarmac road in Tenerife [Additional file 21]. Light pollution from streetlamps disorient young birds when they first leave the nest, causing widespread mortality from traffic collisions and predators. However, as Airam Rodríguez of Estación Biológica de Doñana CSIC explains, there is a more heartwarming side to this story “*To mitigate light pollution-induced mortality rescue campaigns are conducted every fledging season by local governments or NGOs asking for implication of the general public. Thanks to this effort about 90% of rescued birds are successfully released into the wild, giving them a second chance.”*

In a similar vein, at first glance Benjamin P. Y-H. Lee’s aerial photograph of a major highway carving its way through dense forest on the outskirts of Singapore [Additional file 22] appears to be a depressing scene of human encroachment into natural habitats. But look closer and you will see that the bridge spanning the road is a wildlife overpass connecting two rainforest fragments of a nature reserve “*Rainforest afforestation on the overpass with the appropriate plant species will be crucial in forming a functional wildlife corridor between the two fragments. The success of such a mitigation technique can only be shown with the careful planning of monitoring programs (using camera traps and passive ultrasonic recordings) and genetic studies of target animal groups.”*

Photographs such as these serve as a reminder not only of the power of images to convey complex concepts in a visually accessible way, they also offer a small window into the natural world, the lives of ecologists, and the challenges that lay ahead for both.

### Notes

All images published in this Editorial are released under a Creative Commons Attribution License (CC BY) [12] to ensure credit with proper attribution. If you wish to re-distribute or re-use any images published in this Editorial, please credit individual winners as the image licensee.

## Competing interests

SH is an employee of BioMed Central. MB, MBB, DH and JS are Editorial Board Members for *BMC Ecology*. CH declares no competing interests.

## Author’s contributions

SH conceived of the competition and co-wrote the Editorial with guest judge CH. MB, MBB, DH and JS chose category winners and provided quotations for their decisions. All authors read and approved the final manuscript.

## Additional files

## Supplementary Material

Additional file 1: **“Eastern Swallowtails (*Papilio glaucus*) can often be found along river edges in the Eastern U.S. in large numbers.** They will often congregate and feed on mineral deposits on the banks.” Attribution: J.P. Lawrence (University of Mississippi). Click here for file

Additional file 2: **“An anchialine pond named Skippy’s Pond at the ‘Ahihi-Kina’u Natural Area Reserve on South Maui in the Hawaiian Islands.** Anchialine habitats consist of coastal, but landlocked ponds, pools, and caves with subterranean connections to both freshwaters and seawater. On Maui and Hawaii some anchialine habitats such as Skippy’s Pond are characterized by an endemic orange bacterial/cyanobacterial crust community. The striking fluorescent orange of the pond stands out against the stark black lava fields where these habitats are found. Researchers pictured here (Stephanie Irvin and Kiley Seitz) are collecting samples of the crust and shrimp that graze on the crust to determine the crust’s community composition and explore ecological interactions with animals in the anchialine ecosystem” Attribution: Justin Havird (Auburn University). Click here for file

Additional file 3: **“As the sun sets on the Ethiopian Highlands, many animals sense the impending danger from predators that a shift in light conditions brings, scurrying to their protective sleeping sites.** For some animals, like these geladas, the naturally steep descent to their sleeping roosts is too tempting to avoid coupling some acrobatic play with this swift retreat.” Attribution: Ryan J. Burke (University of Oxford). Click here for file

Additional file 4: **“Adult female-infant wild chimpanzees feeding on*Ficus sur*fruits in Kibale National Park, Uganda.** The infant was one-year old, and he was still breast feeding. However, he has been seen to taste the flesh of red (very ripe) fruits. The picture was taken during my postdoctoral fellowship at Harvard University, during which I studied the relationship between contest competition and the nutritional quality of wild fruits. I made the very first detailed study on chimpanzee’s nutritional ecology with descriptions of behaviors never seen before. Most of my observations were collected directly in the canopy with wild chimpanzees all around. Needless to say that this was the project of my life. Picture taken at 25 m above the ground.” Attribution: Alain Houle (Harvard University). Click here for file

Additional file 5: **“*Sphex latreillei*is a beautiful sphecid wasp that has a very interesting sexual behavior.** The female actively reject males from mating with their legs, but when the females came back from hunting with a prey in their legs, the male takes advantage and violently grab the females in the air and throws it in to the ground, were the female can’t reject the male. The photo was taken in central Chile, and shows a couple of wasp mating while the female is holding a tettigonid cricket. The event last only a few seconds, and after that the female leaves the cricket in a subterranean nest to feed his larvae and then go out to hunt again.” Attribution: Bernardo Segura (University of Chile). Click here for file

Additional file 6: **“Sap-sucking tiny insects, though mostly treated as enemies by farmers and gardeners, play an important role in the food chain as food for several other insects.** In this photograph, these are some newly hatched, each about 2 millimetres in length, along with some unhatched eggs on a leaf of a 5 centimetre long sapling.” Attribution: Souvik Mandal (Indian Institute of Science, Bangalore). Click here for file

Additional file 7: **“Snapped in this photograph is an infant (4 months approx.) Slender loris *Loris lyddekerianus lyddekerianus*perching atop a lantana shrub during our field survey of this highly elusive and shy nocturnal species.** This ‘Endangered’ arboreal species now exist in highly fragmented landscapes along the Eastern ghats range of India threatened primarily, by sheer ignorance of their existence, inefficient management of their habitat and extensive loss of their habitats. The behavior illustrated in this snap is termed,‘parking’ wherein Infants lorises are stationed, sometimes communally, by mother at cryptic locations termed, ‘parking spots’ as they depart to catch insect preys for their hungry offsprings.” Attribution: Sayantan Das (University of Mysore). Click here for file

Additional file 8: **“Spotted Owlets *Athene brama*generally roost in small groups in the hollows of trees or in crevices in rocks or buildings.** In daytime, they rarely go out and at night, they come out to prey upon mostly on small vermin rodents, and occasionally on insects and other small vertebrates. The population of this magnificent hunter is now under threat mainly due to habitat destruction which eventually will affect human population. I had to wait for about one hour to get that snap as, I think, they really don’t like attention from a disturbing agent.” Attribution: Souvik Mandal (Indian Institute of Science, Bangalore). Click here for file

Additional file 9: **“An Arctic Hare sprinting across the difficult terrain of the Greenland tundra.** The photos was taken at then end of summer and this individual has started growing its white winter coat. Southwest Greenland.” Attribution: Daniel W. Carstensen (UNESP, Rio Claro, Brazil). Click here for file

Additional file 10: **“Oriental garden lizards, are widely distributed in Asia, eat mainly insects and small vertebrates, including rodents and other lizards.”** Attribution: Anandbabu.R (Pondicherry University). Click here for file

Additional file 11: **“The picture shows*Daphnia magna*, the largest representative of the genus Daphnia, small freshwater crustaceans (also known as water fleas), which are among the oldest model systems in biological research.** Like any other organism, *D. magna* are constantly challenged by microorganisms trying to invade their body. One of these villains is *Pasteuria ramosa*, a bacterial parasite castrating its host after successful establishment. The comparison of a healthy (left) and a heavily infected (right, 30 days post infection) female under a stereomicroscope reveals the consequences of parasite invasion: while the uninfected animal holds developing neonates in its brood chamber, this chamber is empty in the infected animal. Note also, that the hemolymph of the infected female is filled with *P. ramosa* endospores, the transmission stages, hence the opaque instead of translucent appearance of infected compared to healthy females. After the death of the host, these spores will be released from the carcass and consequently be ingested by new hosts, completing the horizontal transmission and starting a new life cycle of *P. ramosa*.” Attribution: Nina Schlotz (University of Konstanz). Click here for file

Additional file 12: **“Two hippos fighting in a shallow water hole in Amboseli National Park, Kenya.** At first we assumed that this was a territorial dispute, but the disparity in size between the two animals and the fact that the aggression was tempered to some extent, led us to wonder if it this behaviour was connected to mating.” Attribution: Graeme Shannon (Colorado State University). Click here for file

Additional file 13: **“The photo was taken during my field work of Ph.D research work on intertidal community structure.** The captured moment shows high competition for space in two different invertebrate community- zoanthid (cnidarian) and Barnacle (arthropod). Zoanthid is colonial cnidarian found in intertidal zone to deep sea water while barnacles are sessile but these both animals are benthos and required space for settlement. The present picture indicates expansion of zoanthids around previously settled live barnacles and they almost covered some small ones. Now, juveniles of barnacles will not get the space for settlement and zoanthid polyps will continuing expand by budding and will covered and grown on all live barnacles.” Attribution: Paresh Poriya (Saurashtra University). Click here for file

Additional file 14: **“A herd of wildebeest in the savannah grassland of Amboseli National Park, Kenya.** These animals are key grazers driving ecosystem dynamics, while also providing an important prey base for the resident lion population.” Attribution: Graeme Shannon (Colorado State University). Click here for file

Additional file 15: **“This image was captured in Pilanesberg National Park, South Africa during a management operation to dart and anesthetize the male lion on the left of the photo.** I managed to take the shot as the lion and lioness tore an adult impala, which was being used as bait, in half. Lions are a key predatory species in the national park that play a crucial ecosystem role. Maintaining a healthy population of lions requires collecting detailed data on genetics, body weight, condition and disease. This image captures both the important management operation and the predatory role and power of lions.” Attribution: Graeme Shannon (Colorado State University). Click here for file

Additional file 16: **“My field of research is focused on the study of long-lived pelagic seabirds. Specifically I am working on Black-browed albatross (*Thelassarche melanophrys*) nesting in dense colonies on New Island (North-west Falkland I.).** My principal objectives are (a) characterize the trophic niche of birds of different ages and breeding status by means the analysis of stable isotope in blood in order to understand trophic interaction during the breeding season; (b) to understand the migration route and off-sea distribution of immature and breeding albatrosses during the wintering season by employing geolocators.” Attribution: Letizia Campioni (Eco-Ethology Research Unit, Portugal). Click here for file

Additional file 17: **“Bats have been identified as important reservoir hosts for pathogens that are able to cross species barriers to infect humans and some species of animals.** Although bats are known to pose a risk to human health, it has been shown that the zoonotic diseases result due to habitat encroachment, bushmeat consumption and urbanization. Long-term disease surveillance programs are needed to understand the viral dynamics in bats and how they impact on human, livestock and wildlife health (“One Health” concept) and how to prevent or curb the outbreak of emerging infectious diseases (EIDs), while ensuring bat species are conserved. This photo shows a field scientist, with the necessary personal protection equipment, releasing a lesser short-nosed fruit bat (*Cynopterus brachyotis*) after taking morphometric measurements and biological samples.” Attribution: Benjamin P. Y-H. Lee (University of Kent). Click here for file

Additional file 18: **“This image is a mixture of five different scenes captured during my field work in coastal areas of western India.** The photos were taken during summer and pre-monsoon of 2013 when I was collecting data for my Ph.D research work. During this period, fishing activities are banned in open ocean due to pre-monsoon activities for safety of fisherman. The peoples in coastal area have to depend on other food resources like molluscs and other invertebrates found in intertidal zones during lowest tide. The present photo express their activities and struggle for food in these seasons.” Attribution: Paresh Poriya (Saurashtra University). Click here for file

Additional file 19: **“Human population and industrialisation is growing at frantic pace.** As a result development is the priority of every developing country, biodiversity conservation concept is secondary in this scenario, when building, township and development is undertaken, the new building and landscaped gardens which are being constructed are not at all friendly for nest formation for birds. The modern glass-clad match box shaped buildings do not have cavities which are very important for nests formation. In today’s perspective humans become as intolerant as a species. For an example, today people do not like birds to make nest in their home or dropping nesting material inside home. Ground water become contaminated with Heavy metals which are very toxic for survival of living beings, As a habitat and food resources are shrinking for birds, we have to take initiatives to protect their habitat and make a new habitat for them to breed.” Attribution: Nagendra Rai (Indian Institute of Toxicology Research). Click here for file

Additional file 20: **“An army of soldier crabs (*Mictyris longicarpus*) marches through (as it must appear to them) a forest of aerial roots of the mangrove*Avicennia marina.*** Soldier crabs roam across mudflats, feeding on detritus and the microphytobenthos. They spend the majority of their time buried in the sand, appearing at low tides to form roaming groups. Due to their behavioural and feeding ecology, soldier crabs can potentially serve as important biomarkers. An analysis of soldier crab physiology can provide information on heavy metal pollution in compromised waterways. This will prove especially useful when conventional water sampling methods cannot detect low concentrations of pollutants.” Attribution: Matthew Nitschke (University of Queensland). Click here for file

Additional file 21: **“One of the most critical phases in the life of a nesting-burrow petrel is fledging.** At this moment, birds have to leave their nests, where they were born and grown, and fly for first time to sea normally at night. Unfortunately thousands of fledglings are disoriented by artificial lights around the world in archipelagos as Hawaii, Azores or Canary Islands. Once birds are grounded they are unable to take off again and susceptible to death by vehicle collisions, starvation, dehydration or predation by introduced predators. To mitigate light pollution-induced mortality rescue campaigns are conducted every fledging season by local governments or NGOs asking for implication of the general public. Thanks to this effort about 90% of rescued birds are successfully released into the wild, giving them a second chance. This Cory’s shearwater *Calonectris diomedea* fledgling picture was taken while researching factors of light pollution-induced mortality in Tenerife, Canary Islands.” Attribution: Airam Rodríguez (Estación Biológica de Doñana CSIC). Click here for file

Additional file 22: **“Wildlife overpasses are used as a mitigation measure worldwide to reduce the mortality of wildlife on roads, and to a certain extent, to facilitate the genetic exchange of both flora and fauna species in forest fragments.** This photo depicts a newly constructed wildlife overpass in highly urbanized Singapore, which connects two rainforest nature reserves that was separated by an eight-laned highway for close to 30 years. Rainforest afforestation on the overpass with the appropriate plant species will be crucial in forming a functional wildlife corridor between the two fragments. The success of such a mitigation technique can only be shown with the careful planning of monitoring programs (using camera traps and passive ultrasonic recordings) and genetic studies of target animal groups.” Attribution: Benjamin P. Y-H. Lee (University of Kent). Click here for file
