# Supplementary figures and images for: BMC Ecology image competition 2014: the winning images
Source: BMC Ecol. 2014 Aug 29;14:24. doi: 10.1186/s12898-014-0024-6 (PMC4236560; doi:10.1186/s12898-014-0024-6)

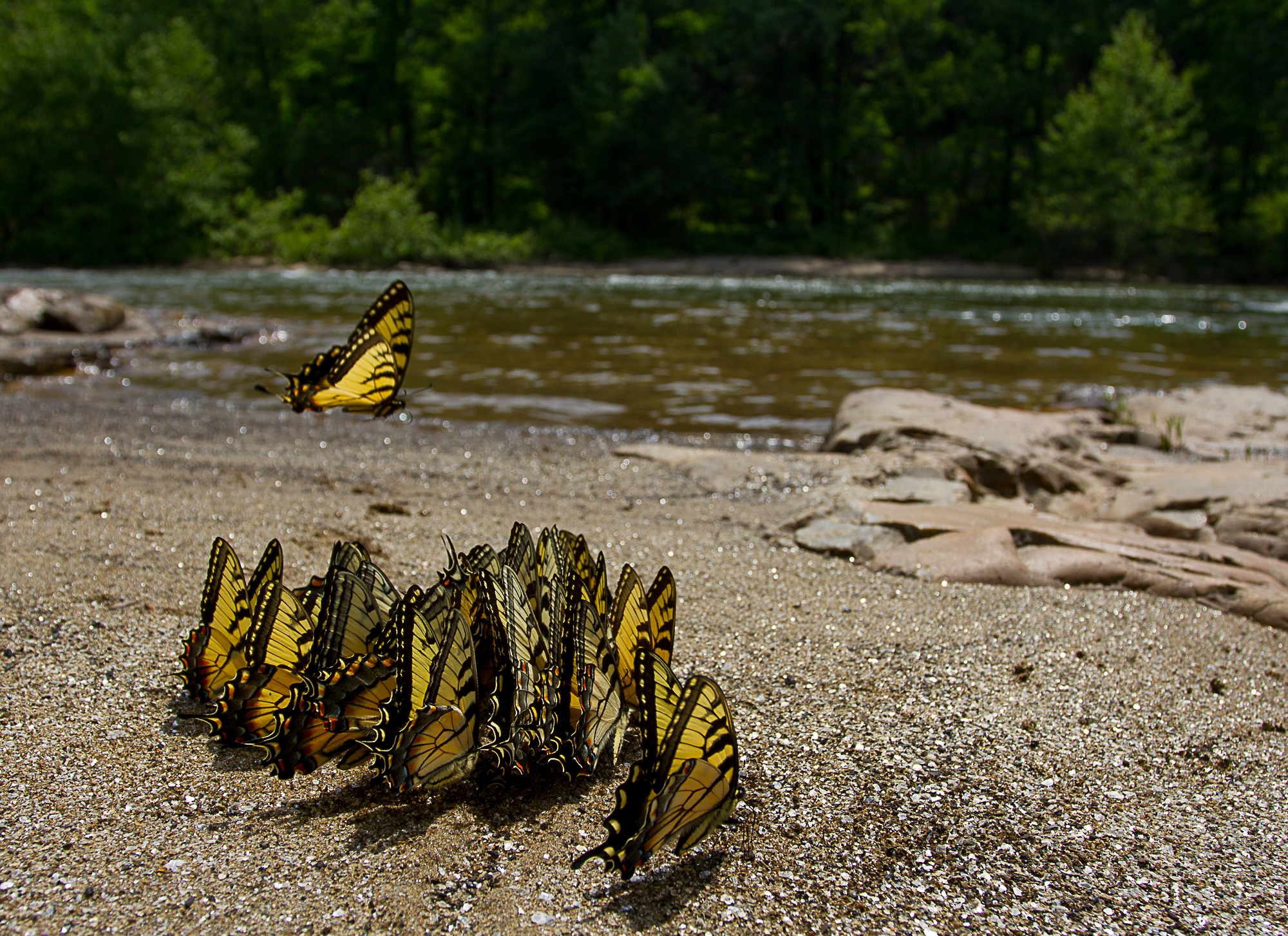

Supplement: Additional file 1: — “Eastern Swallowtails (Papilio glaucus) can often be found along river edges in the Eastern U.S. in large numbers. They will often congregate and feed on mineral deposits on the banks.” Attribution: J.P. Lawrence (University of Mississippi). [file s12898-014-0024-6-S1.jpeg]

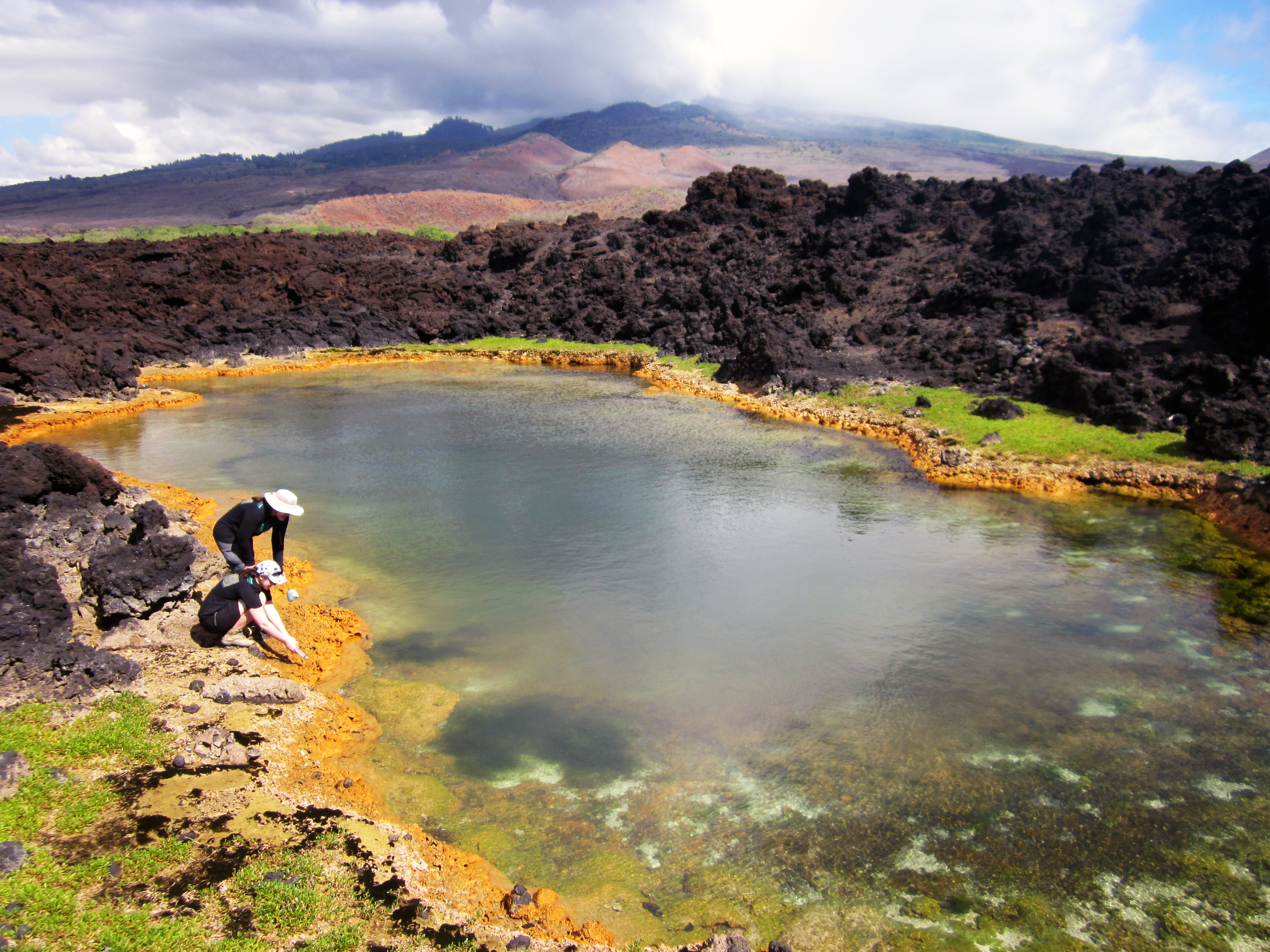

Supplement: Additional file 2: — “An anchialine pond named Skippy’s Pond at the ‘Ahihi-Kina’u Natural Area Reserve on South Maui in the Hawaiian Islands. Anchialine habitats consist of coastal, but landlocked ponds, pools, and caves with subterranean connections to both freshwaters and seawater. On Maui and Hawaii some anchialine habitats such as Skippy’s Pond are characterized by an endemic orange bacterial/cyanobacterial crust community. The striking fluorescent orange of the pond stands out against the stark black lava fields where these habitats are found. Researchers pictured here (Stephanie Irvin and Kiley Seitz) are collecting samples of the crust and shrimp that graze on the crust to determine the crust’s community composition and explore ecological interactions with animals in the anchialine ecosystem” Attribution: Justin Havird (Auburn University). [file s12898-014-0024-6-S2.jpeg]

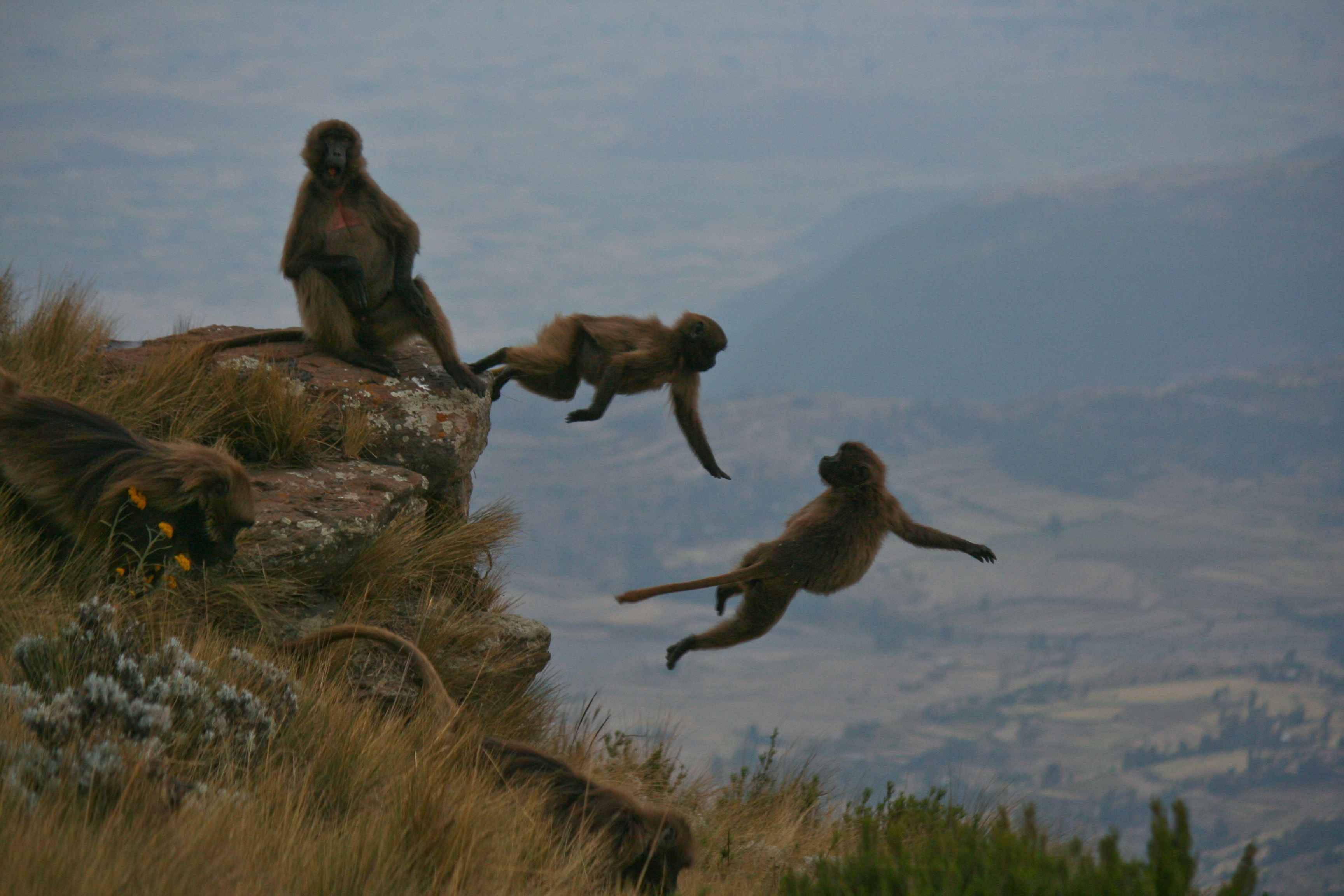

Supplement: Additional file 3: — “As the sun sets on the Ethiopian Highlands, many animals sense the impending danger from predators that a shift in light conditions brings, scurrying to their protective sleeping sites. For some animals, like these geladas, the naturally steep descent to their sleeping roosts is too tempting to avoid coupling some acrobatic play with this swift retreat.” Attribution: Ryan J. Burke (University of Oxford). [file s12898-014-0024-6-S3.jpeg]

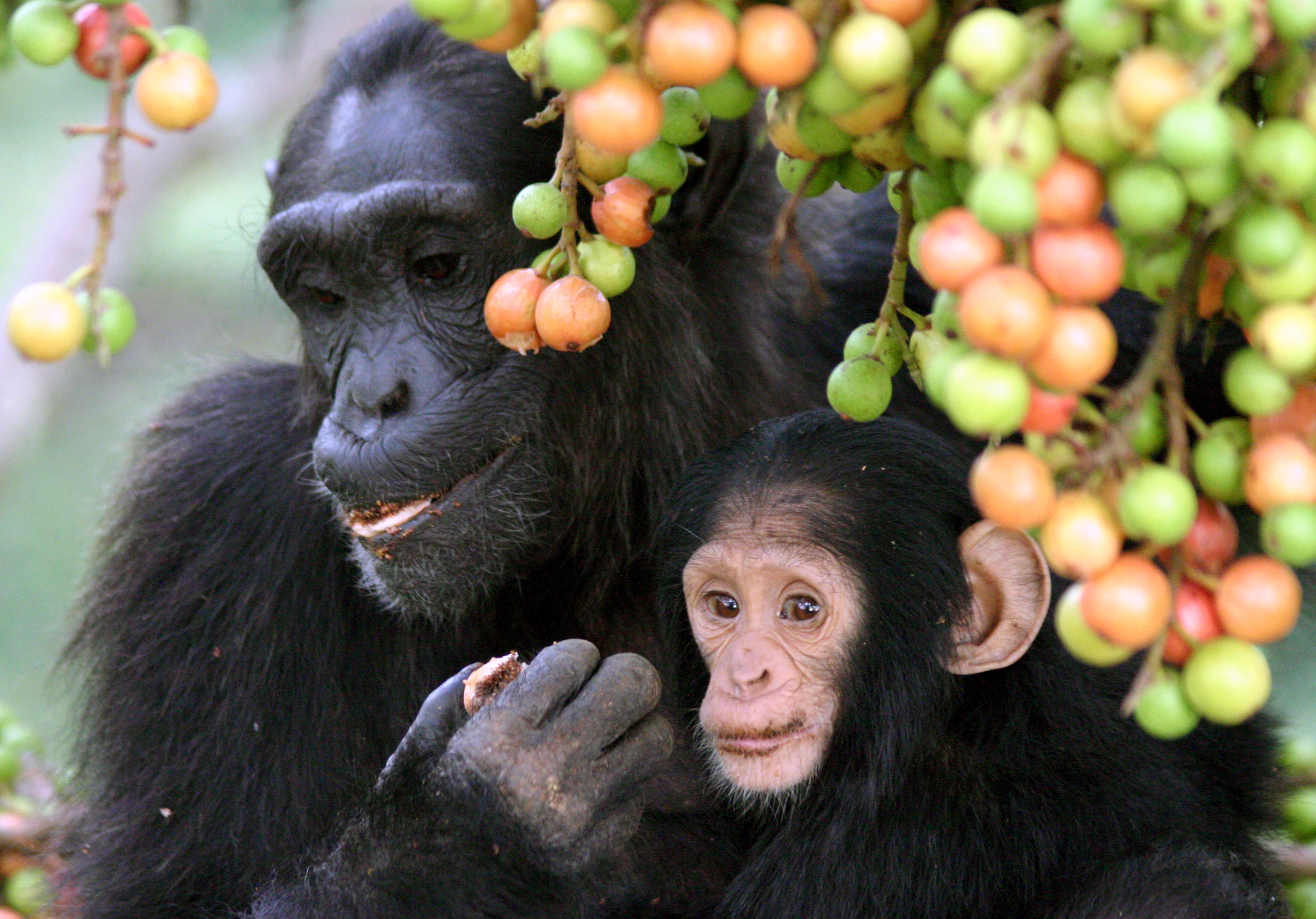

Supplement: Additional file 4: — “Adult female-infant wild chimpanzees feeding onFicus surfruits in Kibale National Park, Uganda. The infant was one-year old, and he was still breast feeding. However, he has been seen to taste the flesh of red (very ripe) fruits. The picture was taken during my postdoctoral fellowship at Harvard University, during which I studied the relationship between contest competition and the nutritional quality of wild fruits. I made the very first detailed study on chimpanzee’s nutritional ecology with descriptions of behaviors never seen before. Most of my observations were collected directly in the canopy with wild chimpanzees all around. Needless to say that this was the project of my life. Picture taken at 25 m above the ground.” Attribution: Alain Houle (Harvard University). [file s12898-014-0024-6-S4.jpeg]

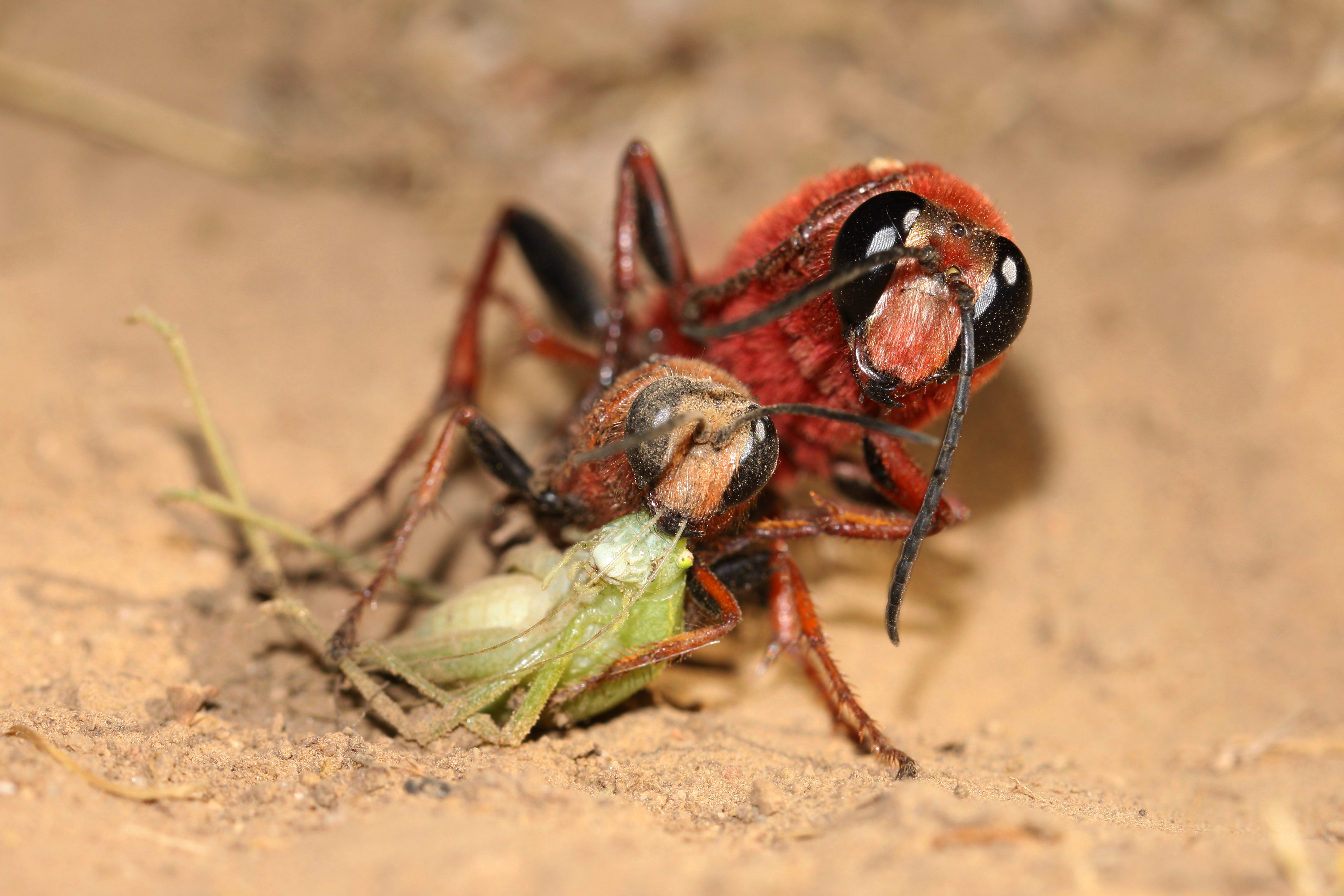

Supplement: Additional file 5: — “Sphex latreilleiis a beautiful sphecid wasp that has a very interesting sexual behavior. The female actively reject males from mating with their legs, but when the females came back from hunting with a prey in their legs, the male takes advantage and violently grab the females in the air and throws it in to the ground, were the female can’t reject the male. The photo was taken in central Chile, and shows a couple of wasp mating while the female is holding a tettigonid cricket. The event last only a few seconds, and after that the female leaves the cricket in a subterranean nest to feed his larvae and then go out to hunt again.” Attribution: Bernardo Segura (University of Chile). [file s12898-014-0024-6-S5.jpeg]

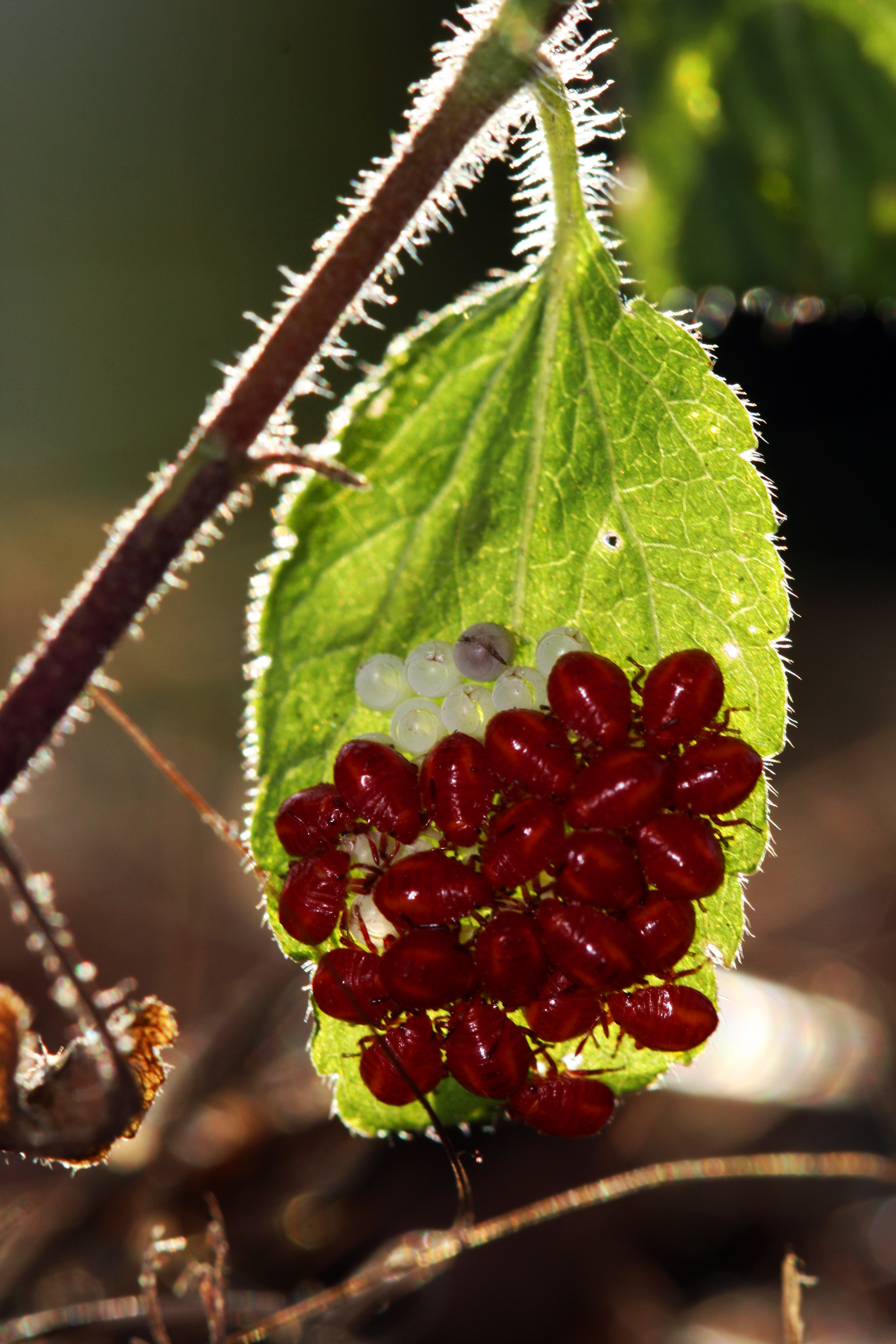

Supplement: Additional file 6: — “Sap-sucking tiny insects, though mostly treated as enemies by farmers and gardeners, play an important role in the food chain as food for several other insects. In this photograph, these are some newly hatched, each about 2 millimetres in length, along with some unhatched eggs on a leaf of a 5 centimetre long sapling.” Attribution: Souvik Mandal (Indian Institute of Science, Bangalore). [file s12898-014-0024-6-S6.jpeg]

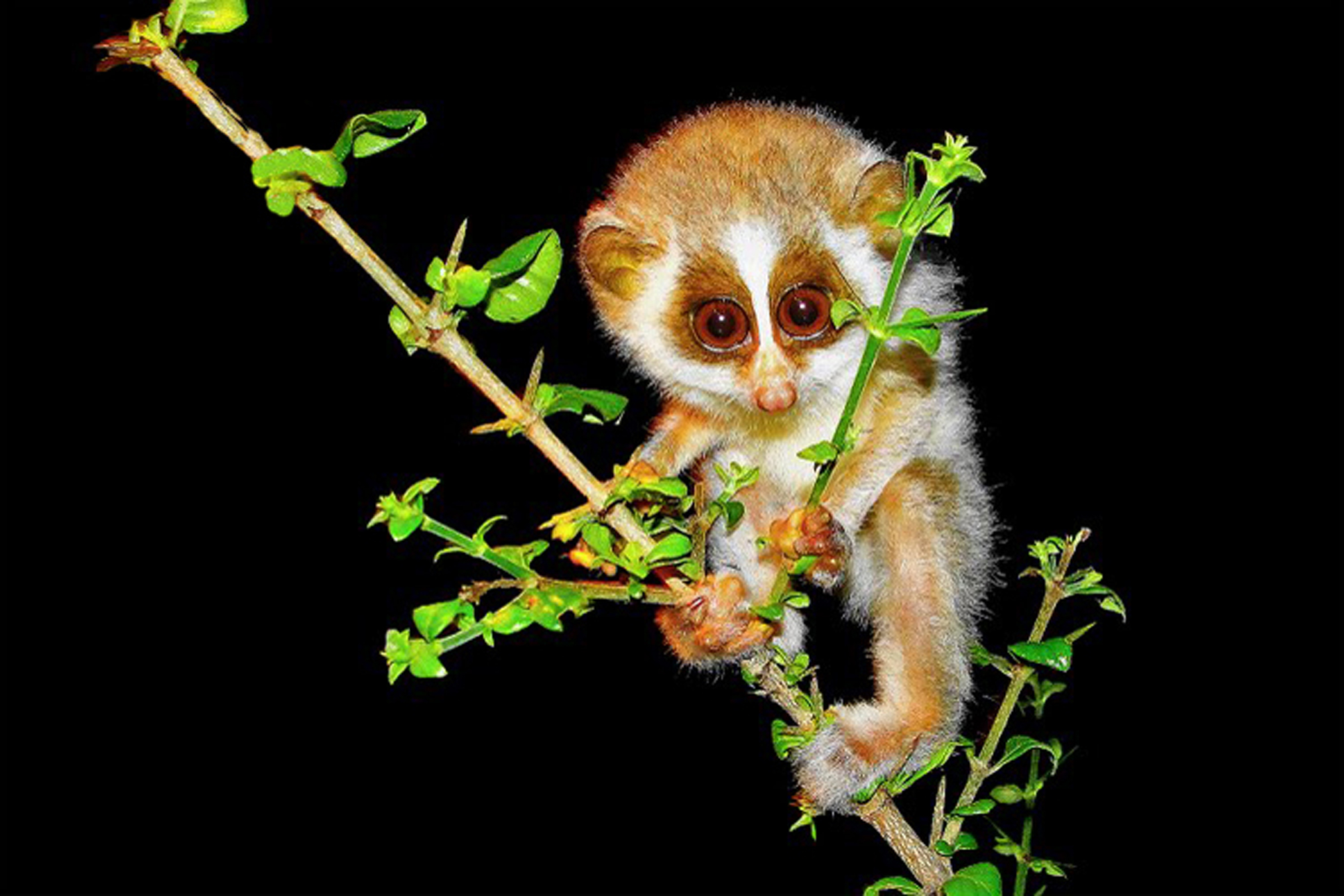

Supplement: Additional file 7: — “Snapped in this photograph is an infant (4 months approx.) Slender loris Loris lyddekerianus lyddekerianusperching atop a lantana shrub during our field survey of this highly elusive and shy nocturnal species. This ‘Endangered’ arboreal species now exist in highly fragmented landscapes along the Eastern ghats range of India threatened primarily, by sheer ignorance of their existence, inefficient management of their habitat and extensive loss of their habitats. The behavior illustrated in this snap is termed,‘parking’ wherein Infants lorises are stationed, sometimes communally, by mother at cryptic locations termed, ‘parking spots’ as they depart to catch insect preys for their hungry offsprings.” Attribution: Sayantan Das (University of Mysore). [file s12898-014-0024-6-S7.jpeg]

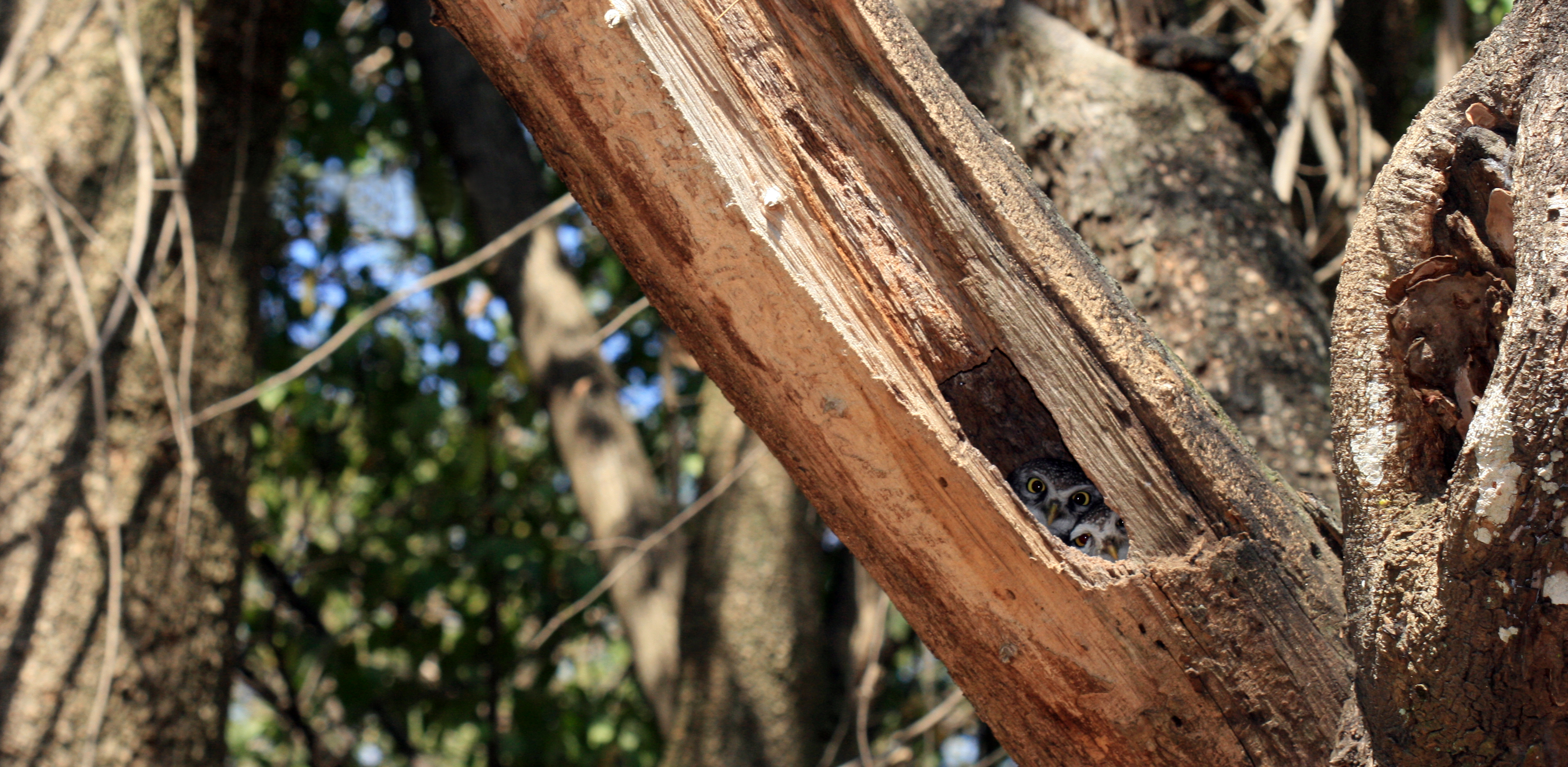

Supplement: Additional file 8: — “Spotted Owlets Athene bramagenerally roost in small groups in the hollows of trees or in crevices in rocks or buildings. In daytime, they rarely go out and at night, they come out to prey upon mostly on small vermin rodents, and occasionally on insects and other small vertebrates. The population of this magnificent hunter is now under threat mainly due to habitat destruction which eventually will affect human population. I had to wait for about one hour to get that snap as, I think, they really don’t like attention from a disturbing agent.” Attribution: Souvik Mandal (Indian Institute of Science, Bangalore). [file s12898-014-0024-6-S8.jpeg]

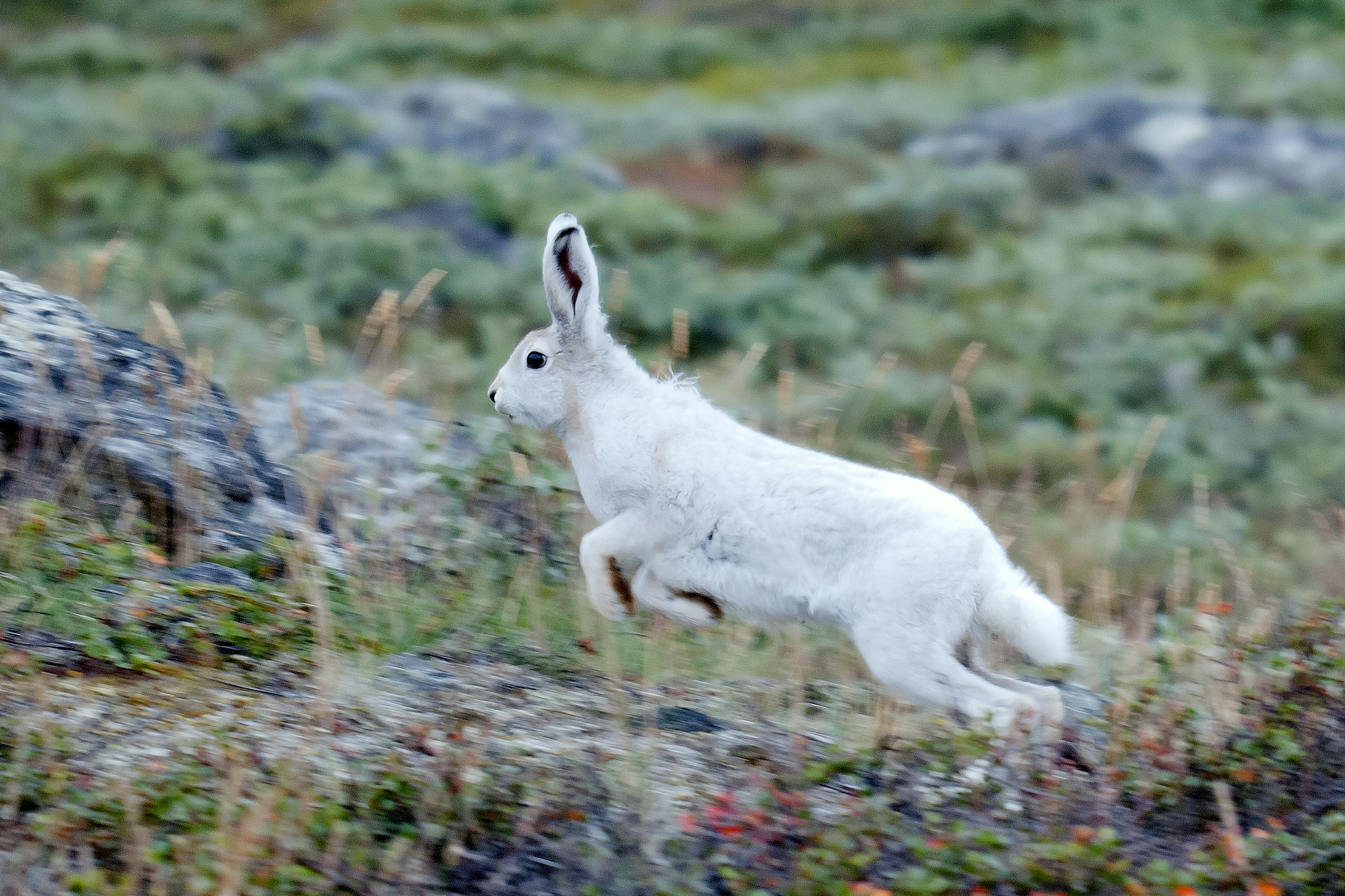

Supplement: Additional file 9: — “An Arctic Hare sprinting across the difficult terrain of the Greenland tundra. The photos was taken at then end of summer and this individual has started growing its white winter coat. Southwest Greenland.” Attribution: Daniel W. Carstensen (UNESP, Rio Claro, Brazil). [file s12898-014-0024-6-S9.jpeg]

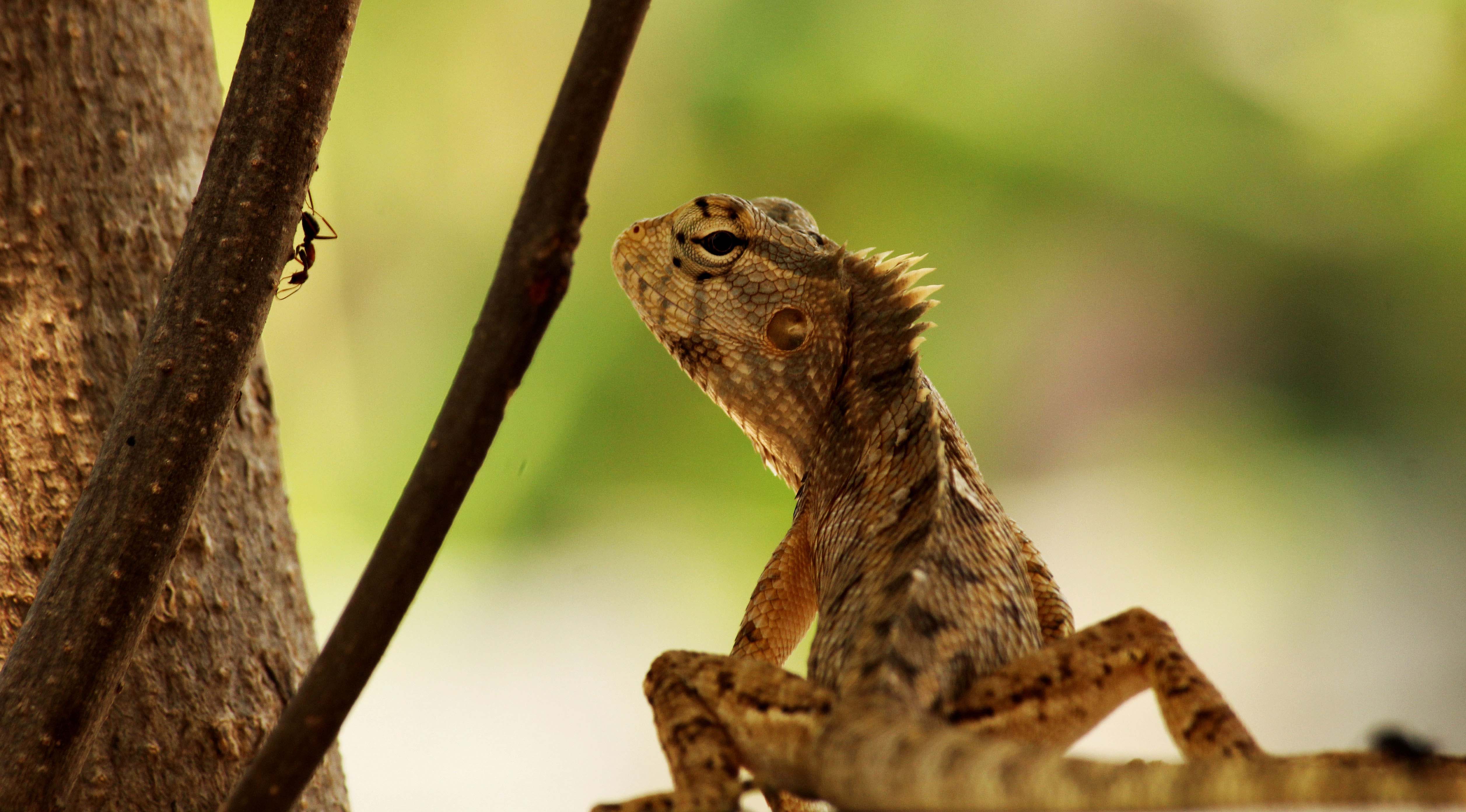

Supplement: Additional file 10: — “Oriental garden lizards, are widely distributed in Asia, eat mainly insects and small vertebrates, including rodents and other lizards.” Attribution: Anandbabu.R (Pondicherry University). [file s12898-014-0024-6-S10.jpeg]

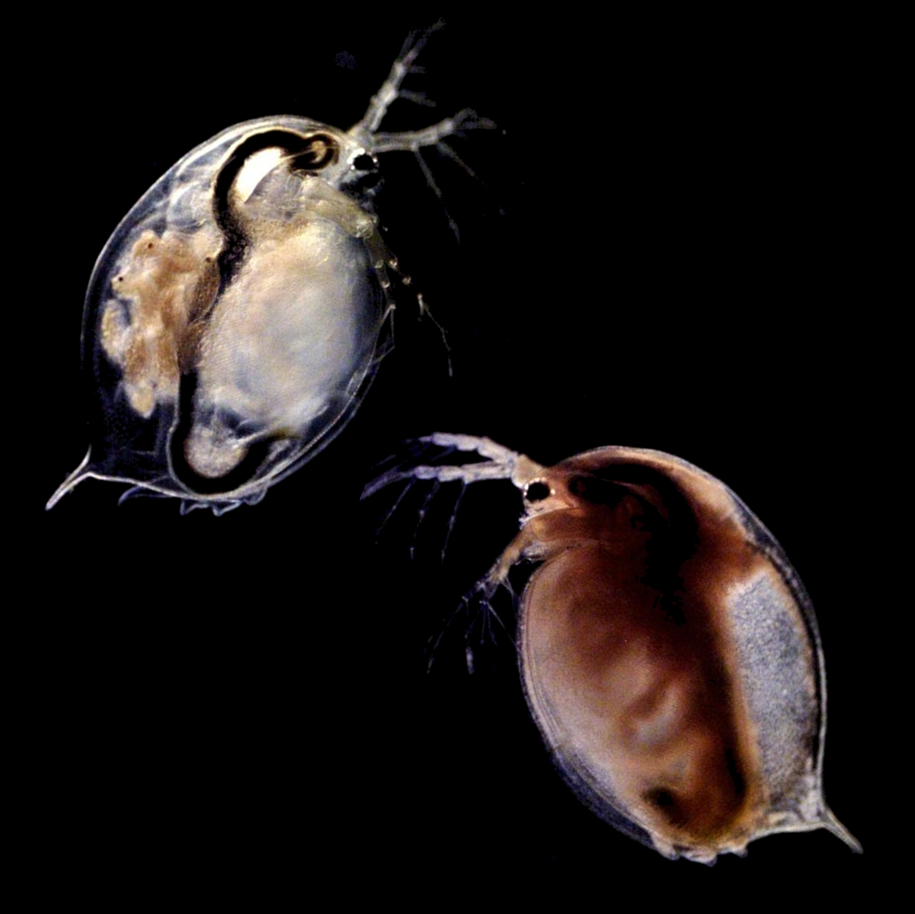

Supplement: Additional file 11: — “The picture showsDaphnia magna, the largest representative of the genus Daphnia, small freshwater crustaceans (also known as water fleas), which are among the oldest model systems in biological research. Like any other organism, D. magna are constantly challenged by microorganisms trying to invade their body. One of these villains is Pasteuria ramosa, a bacterial parasite castrating its host after successful establishment. The comparison of a healthy (left) and a heavily infected (right, 30 days post infection) female under a stereomicroscope reveals the consequences of parasite invasion: while the uninfected animal holds developing neonates in its brood chamber, this chamber is empty in the infected animal. Note also, that the hemolymph of the infected female is filled with P. ramosa endospores, the transmission stages, hence the opaque instead of translucent appearance of infected compared to healthy females. After the death of the host, these spores will be released from the carcass and consequently be ingested by new hosts, completing the horizontal transmission and starting a new life cycle of P. ramosa.” Attribution: Nina Schlotz (University of Konstanz). [file s12898-014-0024-6-S11.tiff]

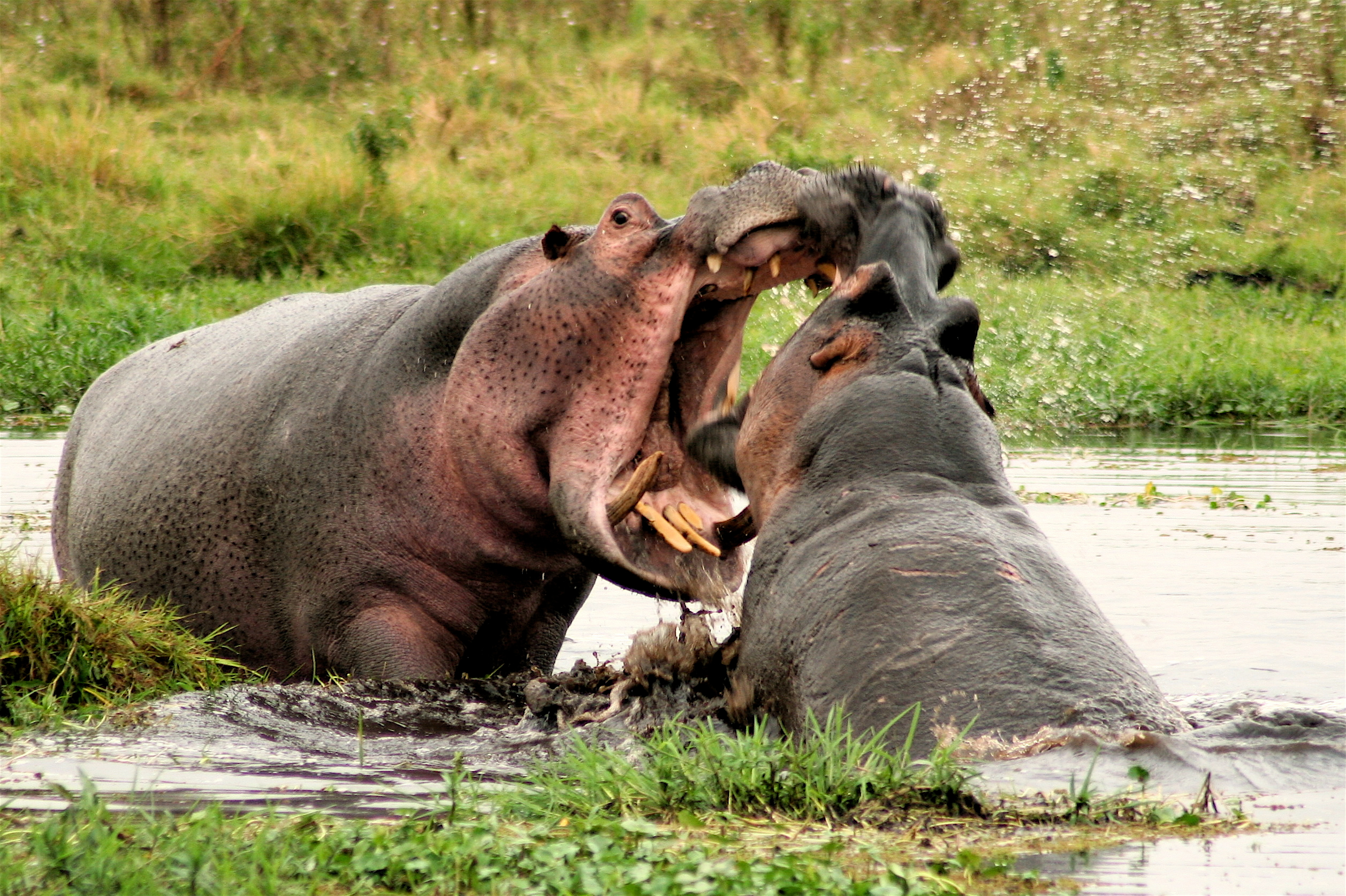

Supplement: Additional file 12: — “Two hippos fighting in a shallow water hole in Amboseli National Park, Kenya. At first we assumed that this was a territorial dispute, but the disparity in size between the two animals and the fact that the aggression was tempered to some extent, led us to wonder if it this behaviour was connected to mating.” Attribution: Graeme Shannon (Colorado State University). [file s12898-014-0024-6-S12.jpeg]

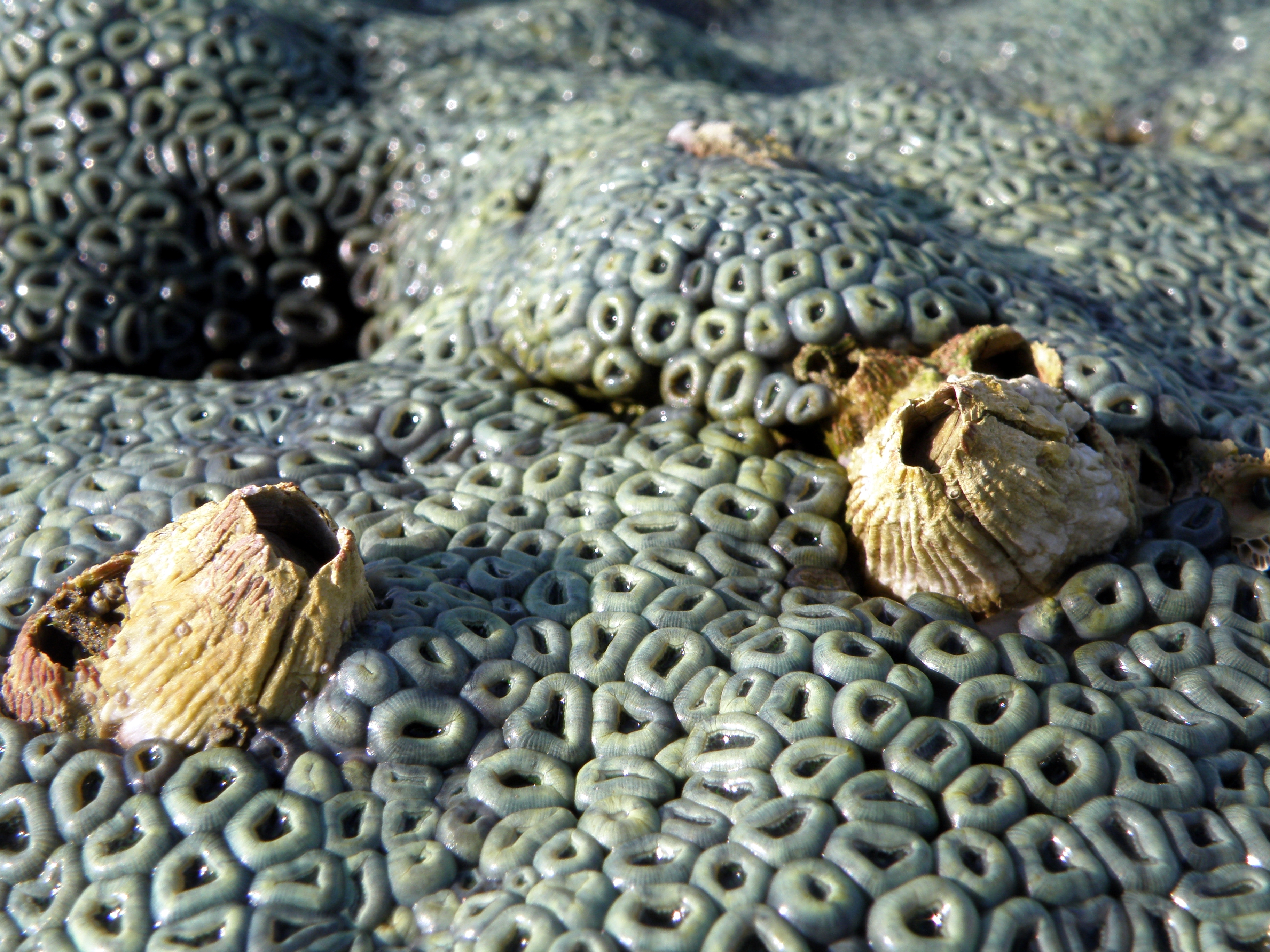

Supplement: Additional file 13: — “The photo was taken during my field work of Ph.D research work on intertidal community structure. The captured moment shows high competition for space in two different invertebrate community- zoanthid (cnidarian) and Barnacle (arthropod). Zoanthid is colonial cnidarian found in intertidal zone to deep sea water while barnacles are sessile but these both animals are benthos and required space for settlement. The present picture indicates expansion of zoanthids around previously settled live barnacles and they almost covered some small ones. Now, juveniles of barnacles will not get the space for settlement and zoanthid polyps will continuing expand by budding and will covered and grown on all live barnacles.” Attribution: Paresh Poriya (Saurashtra University). [file s12898-014-0024-6-S13.jpeg]

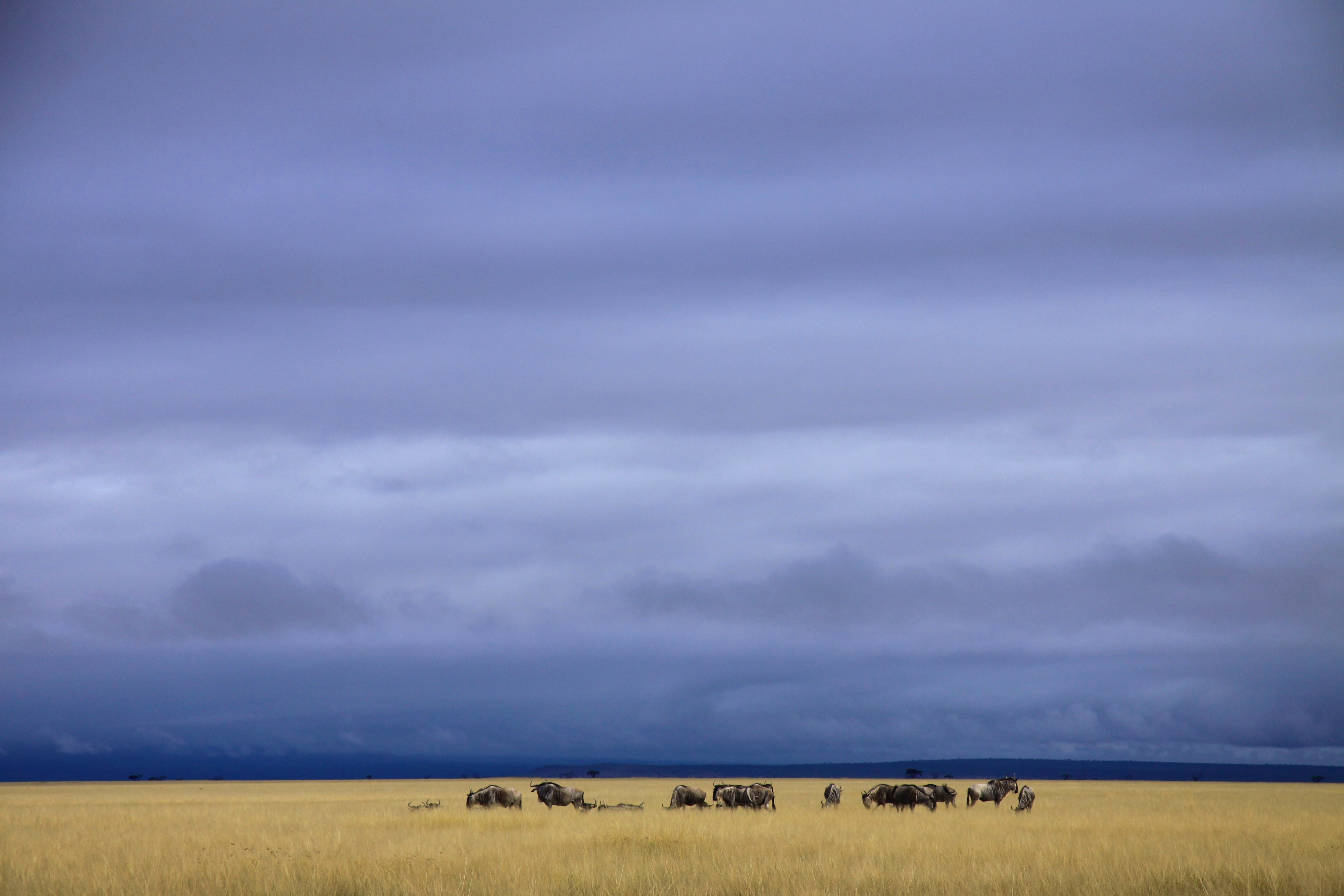

Supplement: Additional file 14: — “A herd of wildebeest in the savannah grassland of Amboseli National Park, Kenya. These animals are key grazers driving ecosystem dynamics, while also providing an important prey base for the resident lion population.” Attribution: Graeme Shannon (Colorado State University). [file s12898-014-0024-6-S14.jpeg]

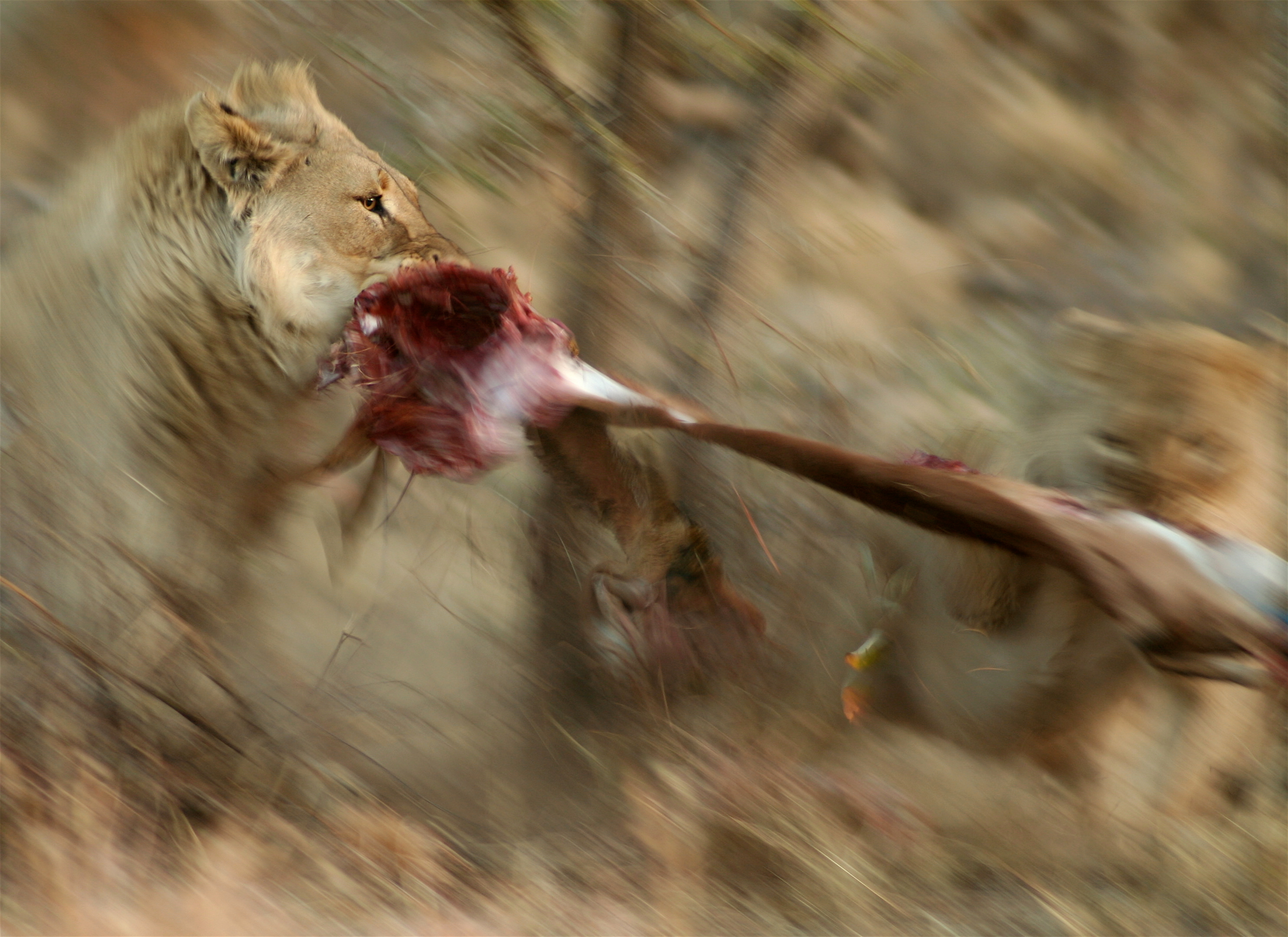

Supplement: Additional file 15: — “This image was captured in Pilanesberg National Park, South Africa during a management operation to dart and anesthetize the male lion on the left of the photo. I managed to take the shot as the lion and lioness tore an adult impala, which was being used as bait, in half. Lions are a key predatory species in the national park that play a crucial ecosystem role. Maintaining a healthy population of lions requires collecting detailed data on genetics, body weight, condition and disease. This image captures both the important management operation and the predatory role and power of lions.” Attribution: Graeme Shannon (Colorado State University). [file s12898-014-0024-6-S15.jpeg]

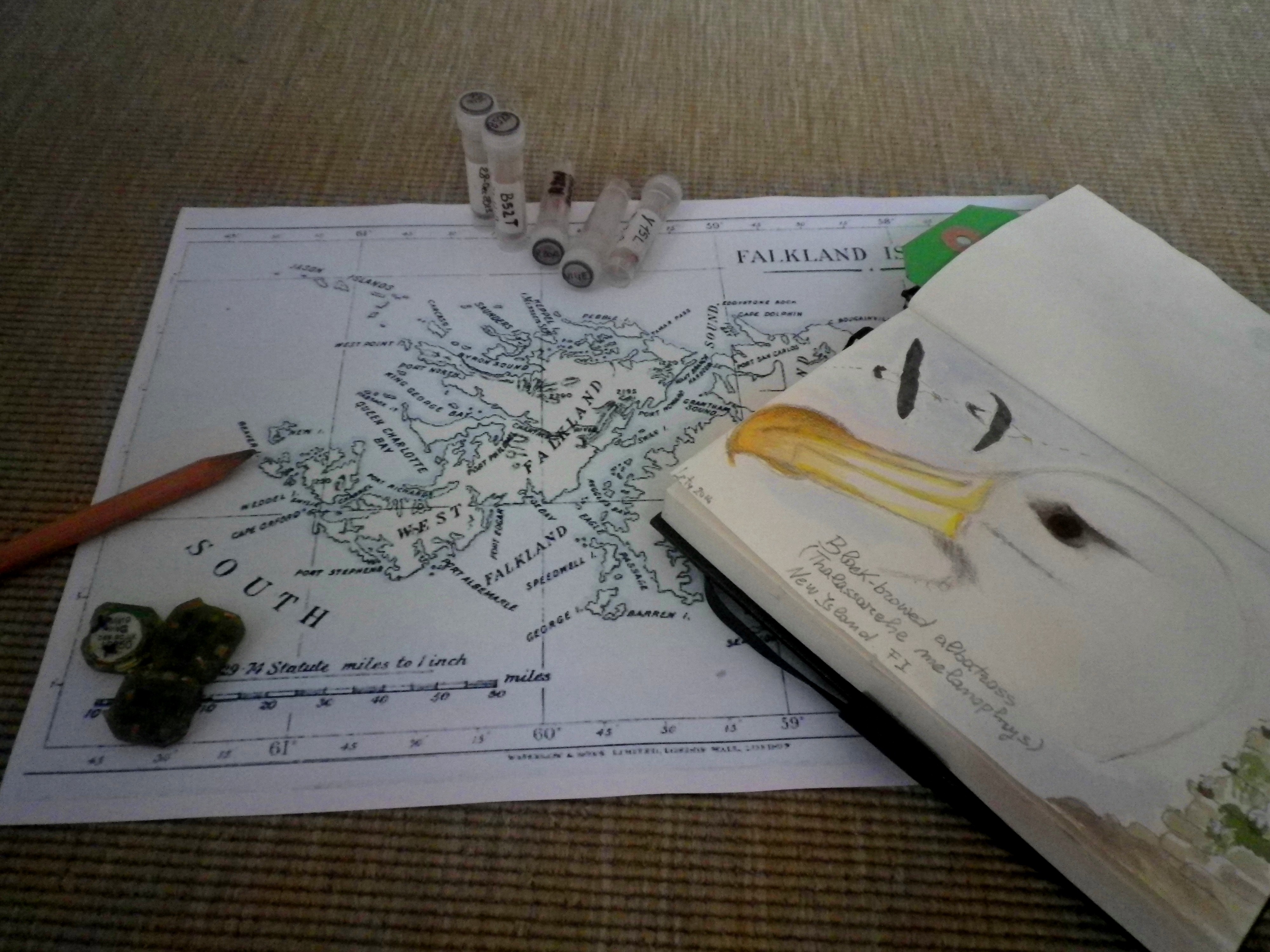

Supplement: Additional file 16: — “My field of research is focused on the study of long-lived pelagic seabirds. Specifically I am working on Black-browed albatross (Thelassarche melanophrys) nesting in dense colonies on New Island (North-west Falkland I.). My principal objectives are (a) characterize the trophic niche of birds of different ages and breeding status by means the analysis of stable isotope in blood in order to understand trophic interaction during the breeding season; (b) to understand the migration route and off-sea distribution of immature and breeding albatrosses during the wintering season by employing geolocators.” Attribution: Letizia Campioni (Eco-Ethology Research Unit, Portugal). [file s12898-014-0024-6-S16.jpeg]

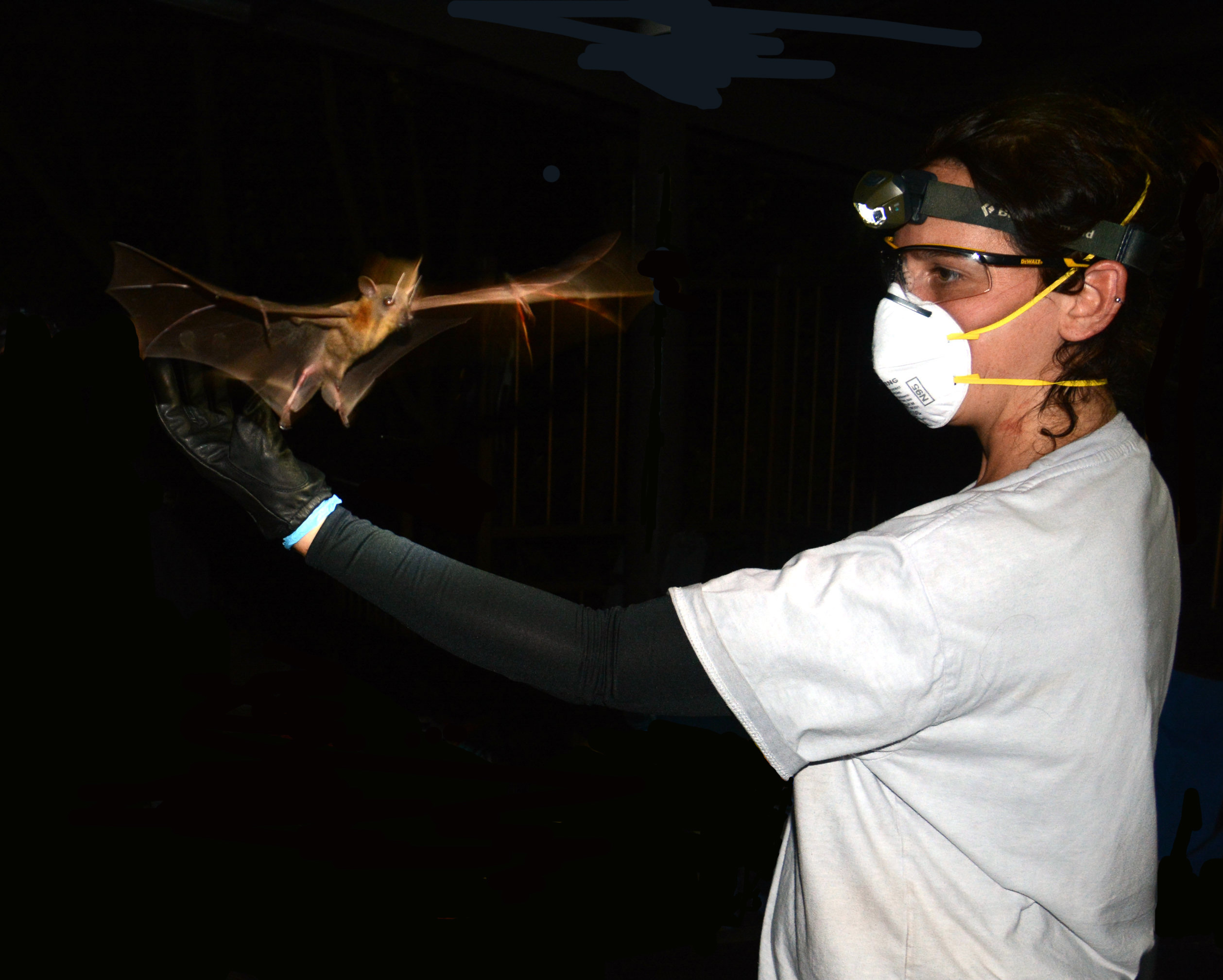

Supplement: Additional file 17: — “Bats have been identified as important reservoir hosts for pathogens that are able to cross species barriers to infect humans and some species of animals. Although bats are known to pose a risk to human health, it has been shown that the zoonotic diseases result due to habitat encroachment, bushmeat consumption and urbanization. Long-term disease surveillance programs are needed to understand the viral dynamics in bats and how they impact on human, livestock and wildlife health (“One Health” concept) and how to prevent or curb the outbreak of emerging infectious diseases (EIDs), while ensuring bat species are conserved. This photo shows a field scientist, with the necessary personal protection equipment, releasing a lesser short-nosed fruit bat (Cynopterus brachyotis) after taking morphometric measurements and biological samples.” Attribution: Benjamin P. Y-H. Lee (University of Kent). [file s12898-014-0024-6-S17.jpeg]

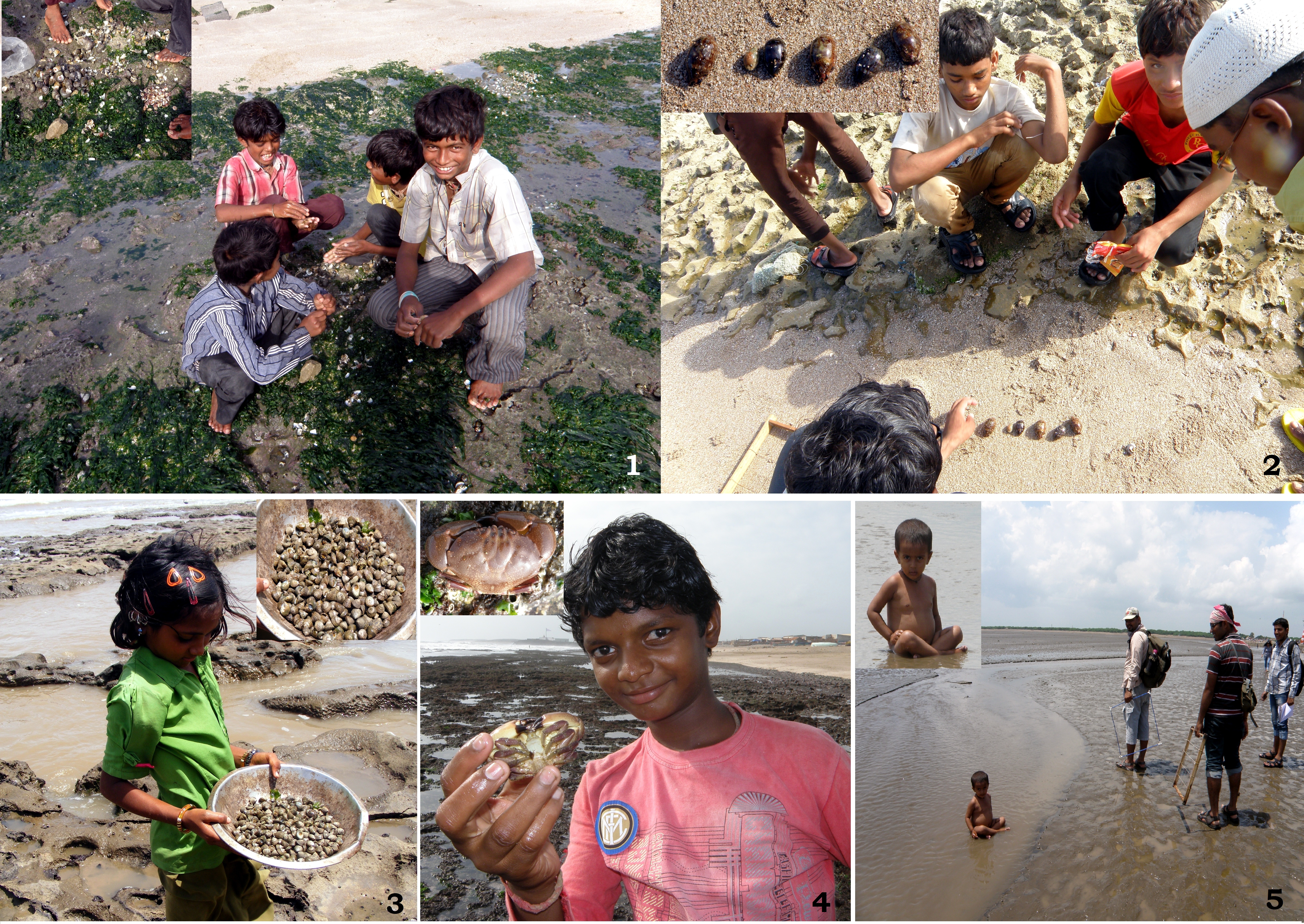

Supplement: Additional file 18: — “This image is a mixture of five different scenes captured during my field work in coastal areas of western India. The photos were taken during summer and pre-monsoon of 2013 when I was collecting data for my Ph.D research work. During this period, fishing activities are banned in open ocean due to pre-monsoon activities for safety of fisherman. The peoples in coastal area have to depend on other food resources like molluscs and other invertebrates found in intertidal zones during lowest tide. The present photo express their activities and struggle for food in these seasons.” Attribution: Paresh Poriya (Saurashtra University). [file s12898-014-0024-6-S18.jpeg]

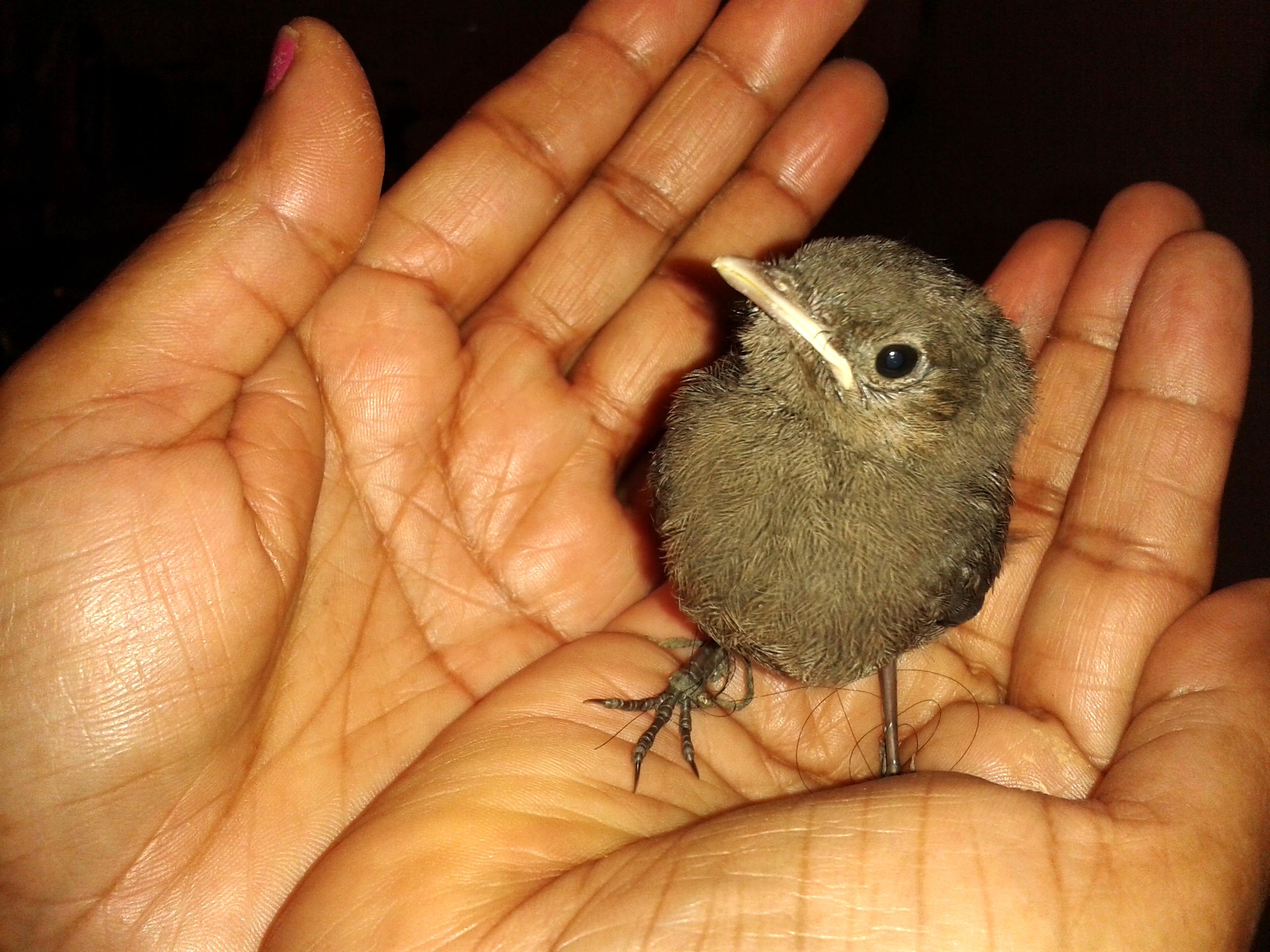

Supplement: Additional file 19: — “Human population and industrialisation is growing at frantic pace. As a result development is the priority of every developing country, biodiversity conservation concept is secondary in this scenario, when building, township and development is undertaken, the new building and landscaped gardens which are being constructed are not at all friendly for nest formation for birds. The modern glass-clad match box shaped buildings do not have cavities which are very important for nests formation. In today’s perspective humans become as intolerant as a species. For an example, today people do not like birds to make nest in their home or dropping nesting material inside home. Ground water become contaminated with Heavy metals which are very toxic for survival of living beings, As a habitat and food resources are shrinking for birds, we have to take initiatives to protect their habitat and make a new habitat for them to breed.” Attribution: Nagendra Rai (Indian Institute of Toxicology Research). [file s12898-014-0024-6-S19.jpeg]

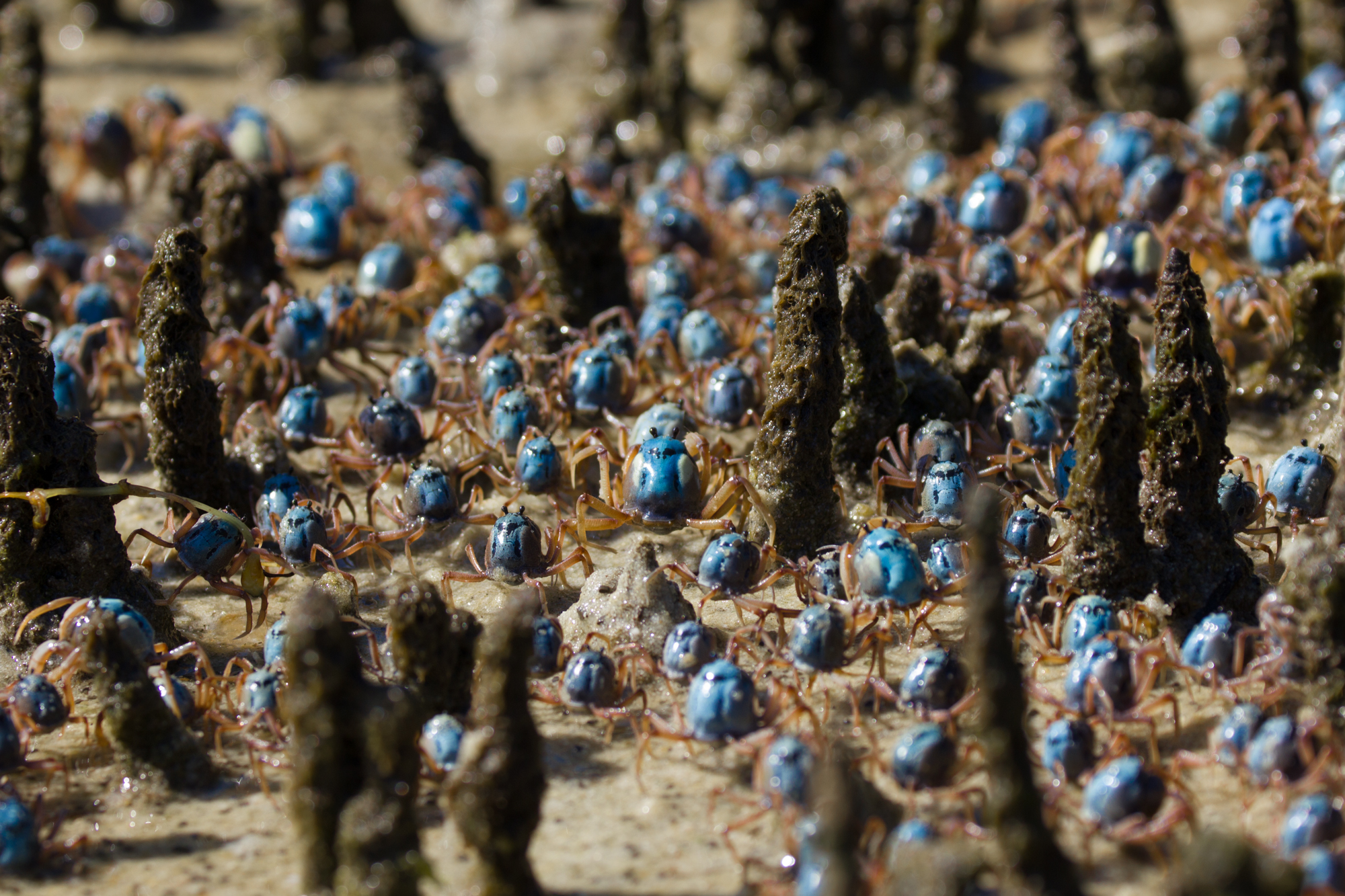

Supplement: Additional file 20: — “An army of soldier crabs (Mictyris longicarpus) marches through (as it must appear to them) a forest of aerial roots of the mangroveAvicennia marina. Soldier crabs roam across mudflats, feeding on detritus and the microphytobenthos. They spend the majority of their time buried in the sand, appearing at low tides to form roaming groups. Due to their behavioural and feeding ecology, soldier crabs can potentially serve as important biomarkers. An analysis of soldier crab physiology can provide information on heavy metal pollution in compromised waterways. This will prove especially useful when conventional water sampling methods cannot detect low concentrations of pollutants.” Attribution: Matthew Nitschke (University of Queensland). [file s12898-014-0024-6-S20.jpeg]

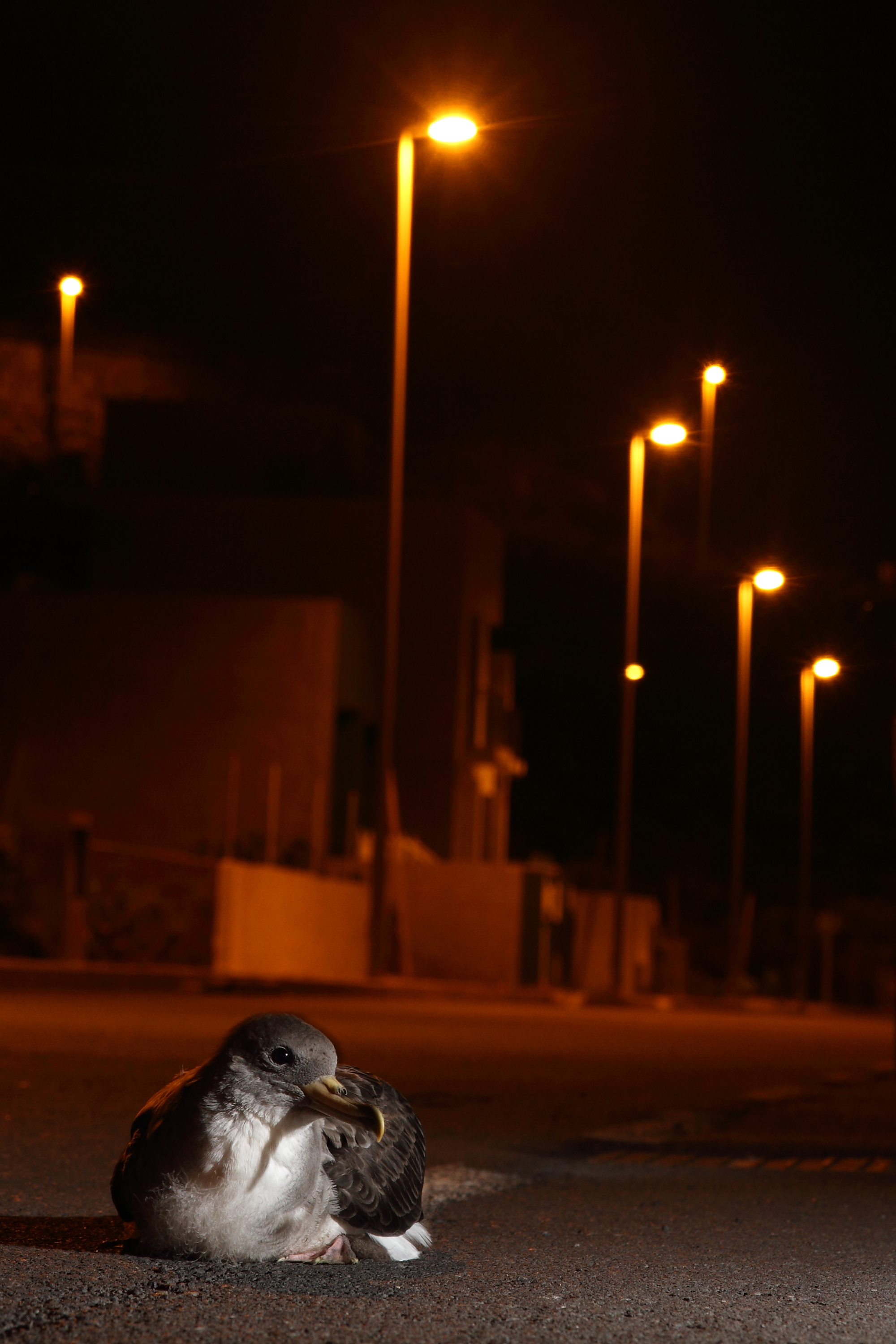

Supplement: Additional file 21: — “One of the most critical phases in the life of a nesting-burrow petrel is fledging. At this moment, birds have to leave their nests, where they were born and grown, and fly for first time to sea normally at night. Unfortunately thousands of fledglings are disoriented by artificial lights around the world in archipelagos as Hawaii, Azores or Canary Islands. Once birds are grounded they are unable to take off again and susceptible to death by vehicle collisions, starvation, dehydration or predation by introduced predators. To mitigate light pollution-induced mortality rescue campaigns are conducted every fledging season by local governments or NGOs asking for implication of the general public. Thanks to this effort about 90% of rescued birds are successfully released into the wild, giving them a second chance. This Cory’s shearwater Calonectris diomedea fledgling picture was taken while researching factors of light pollution-induced mortality in Tenerife, Canary Islands.” Attribution: Airam Rodríguez (Estación Biológica de Doñana CSIC). [file s12898-014-0024-6-S21.jpeg]

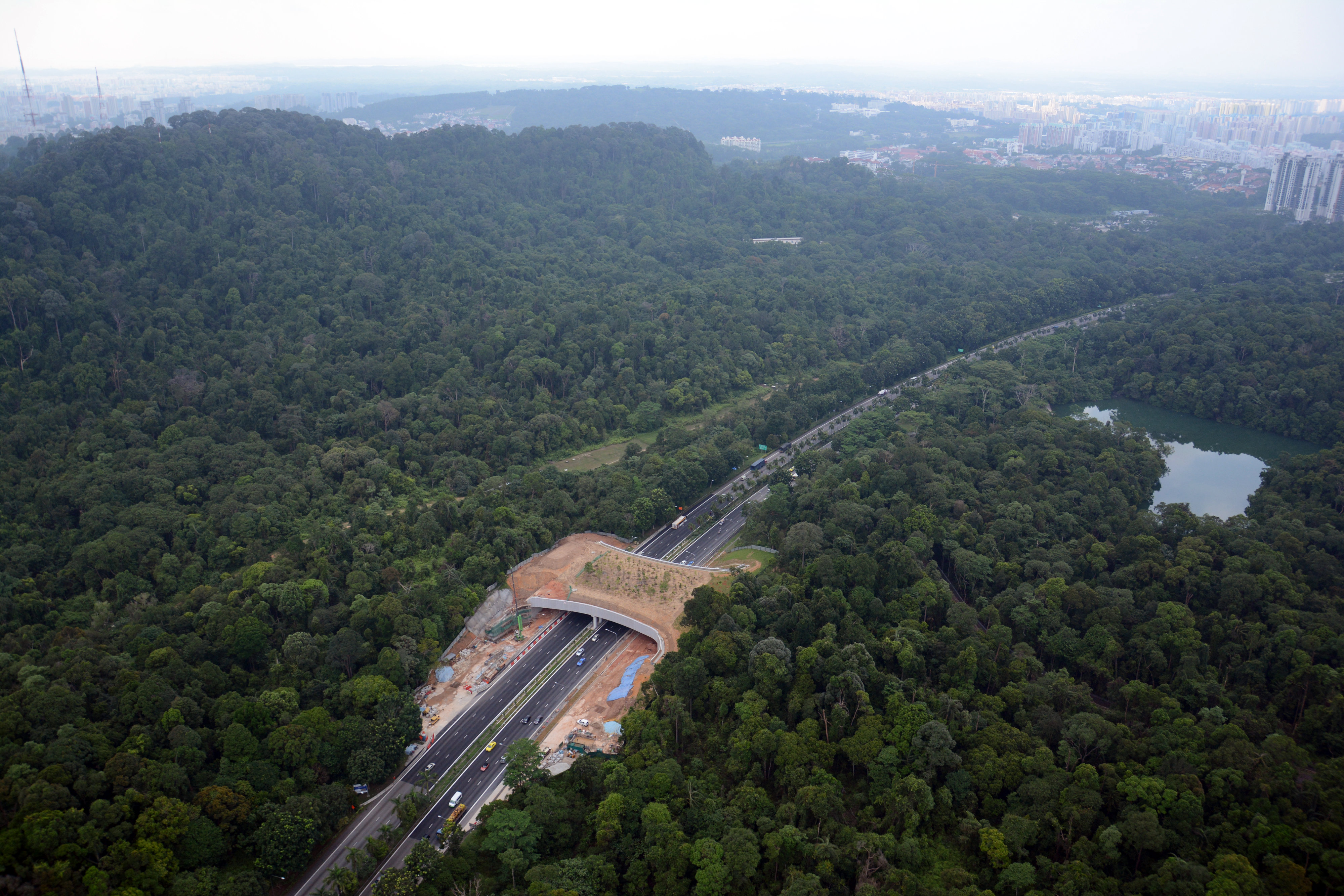

Supplement: Additional file 22: — “Wildlife overpasses are used as a mitigation measure worldwide to reduce the mortality of wildlife on roads, and to a certain extent, to facilitate the genetic exchange of both flora and fauna species in forest fragments. This photo depicts a newly constructed wildlife overpass in highly urbanized Singapore, which connects two rainforest nature reserves that was separated by an eight-laned highway for close to 30 years. Rainforest afforestation on the overpass with the appropriate plant species will be crucial in forming a functional wildlife corridor between the two fragments. The success of such a mitigation technique can only be shown with the careful planning of monitoring programs (using camera traps and passive ultrasonic recordings) and genetic studies of target animal groups.” Attribution: Benjamin P. Y-H. Lee (University of Kent). [file s12898-014-0024-6-S22.jpeg]
